# Transcriptomics Reveal Molecular Signatures of a Resolved Sexual Conflict and Potential Association With Colour Polymorphism in Tawny Owls

**DOI:** 10.1111/mec.70338

**Published:** 2026-04-10

**Authors:** Miguel Baltazar‐Soares, Melanie J. Heckwolf, Marc P. Hoeppner, Patrik Karell, Dominic Wright, Jan‐Åke Nilsson, Jon E. Brommer

**Affiliations:** ^1^ Department of Biology University of Turku Turku Finland; ^2^ Leibniz Center for Tropical Marine Research (ZMT) Bremen Germany; ^3^ Institute of Clinical Molecular Biology, Christian‐Albrechts‐University of Kiel Kiel Germany; ^4^ Department of Biology, Section of Evolutionary Ecology Lund University Lund Sweden; ^5^ Department of Ecology and Genetics University of Uppsala Uppsala Sweden; ^6^ Department of Bioeconomy Novia University of Applied Sciences Tammisaari Finland; ^7^ IFM Biology Linköping University Linköping Sweden

**Keywords:** alternative exon usage, differential expression, sexual conflict, sexual dimorphism, tawny owls, transcriptome

## Abstract

Genome sharing in gonochorous species is expected to result in intraspecific conflicts due to intersexual competition. The emergence of sexual dimorphism is thus connected to the evolution of mechanisms that, starting from a similar genomic background, produce sufficiently disparate phenotypes to attenuate sexually antagonistic selection. From a molecular perspective it can be achieved through sex‐specific differences in gene expression, splicing, non‐coding regulation or epigenetic marks. The tawny owl ( *Strix aluco* ) is a sexually dimorphic species where females and males evolved distinct body sizes (smaller males), which results in sex‐specific roles and therefore is a robust example of resolved sexual conflict. Here, we explore transcriptional variation among 32 juvenile tawny owls with the objective of investigating molecular signatures of resolved sexual conflict. Our results show substantial sex‐specific variation in terms of differentially expressed genes, single nucleotide polymorphisms and alternative exon usage in genes involved in life history traits (*ZGRF1*, *VLDLR*), behaviour (*GSK3B*, *SLC12A*) and aspects of growth (*GHR*, *EGF, EPS8L2*). Exploring sex‐specific DEG revealed enrichment for biological functions associated with melanogenesis and pigment granulation in males, which together with the identification of a single up‐regulated autosomal gene involved in melanogenesis (*RAB38*) in brown males strongly suggests different timings for the onset of pigmentation between sexes. Overall, our results reveal some of the sex‐specific molecular signatures expected to be observed in the context of a resolved sexual conflict.

## 1 Introduction

Sexual dimorphism generally pertains to between‐sex differences both in phenotype or behaviour within a species (Roff and Fairbairn 2007). Perhaps the most relatable example of sexual dimorphism (SD) is body size sexual dimorphism (BSD), where either males or females attain larger body sizes than their counterpart (Tombak et al. 2024). The evolution of sexual dimorphism has been a long‐standing research topic in evolutionary biology due to the challenges in resolving the paradoxical scenario where sexually dimorphic traits are heritable despite the genome being shared by both sexes (Roff and Fairbairn 2007). Such a shared genomic architecture could preclude males and females from reaching their fitness optima, thus constraining the evolution of sexual dimorphism (Wright et al. 2019). It is nowadays accepted that resolved sexual conflicts, and the respective evolution of sexually dimorphic systems, are due to sex‐specific characters being regulated via the sex‐chromosomes (whose expression is mediated by several types of dosage compensation mechanisms) or by sex‐specific gene expression of autosomal genes (Connallon and Knowles 2005). Indeed, transcriptional dimorphism encoding sexually dimorphic traits, or sex‐biased gene expression, has been commonly reported in the autosomes of sexually dimorphic species (Ellegren and Parsch 2007). In addition, sex‐specific exon usage or sex‐specific isoforms have been suggested to have evolved as solutions to intra‐sexual conflict, though research on this specific topic is still in its infancy outside traditional genome model species (Rogers et al. 2021). Our work thus aims to provide molecular evidence of those events. Tawny owls are nocturnal birds of prey (Strigiformes), widely distributed in Europe and Central Asia. Females of this species attain overall larger body sizes than males, and also differences in duty division and breeding roles: females care for eggs and offspring and males acquire food for the female during incubation and for their offspring (Karell et al. 2021). As all birds, tawny owls' sexual chromosomes entail a ZZ/ZW configuration where females are the heterogametic sex. Tawny owls are also characterized by intra‐population melanin‐based colour polymorphism, which results in a bimodal frequency distribution of grey‐ and brown‐plumage individuals (Brommer et al. 2005). Notably, colour morph frequencies were shown to vary locally, with environmental conditions, and recent research shows some support for the frequencies of colour polymorphism within populations to be a signature of local adaptation (Baltazar‐Soares et al. 2024). General patterns of tawny owl plumage colouration are highly heritable (Brommer et al. 2005; Karell et al. 2021) and are caused by irreversible deposition of primarily phaeomelanin pigments in the feathers (Gasparini et al. 2009). Targeted qPCR expression on 9 genes involved in conserved melanin production pathways indeed showed differential expression across the tawny owl colour morphs (Emaresi et al. 2013). Interestingly, Emaresi and colleagues indeed reported extensive age‐, colour‐ and sex‐specific expression patterns (Emaresi et al. 2013), despite the overall patterns of adult plumage colouration remaining independent of bird age and sex.

Our work has two main objectives: the first is to identify molecular signatures of sexual dimorphism on the tawny owl ( *Strix aluco* ) with the clear expectation to shed light on the evolution of sexual dimorphism in general; the second is to explore the relationship between sexual dimorphism and colouration. To accomplish these objectives, we sequenced the full transcriptome from developing feather buds of 32 juvenile tawny owls that lived in a common environment (aviary) and had equal proportions of males and females of both colour morphs, and explored differences both at the gene expression level including sex and colour‐specific alternative splicing, and identified SNPs whose frequencies are fixed within groups. Next, we aimed to explore in‐depth the relationship between sex and colouration beyond the gene‐targeted approach performed by Emaresi et al. (2013). The plausibility to expect such relationships relates to the premise that tawny owl BSD is the outcome of a resolved sexual conflict and thus prone to sex‐specific developmental trajectories for instance, sexual bimaturation is pervasive in species exhibiting body size sexual dimorphism (Stamps and Krishnan 1997; Teder et al. 2021). In line with general expectations of a resolved sexual conflict, we hypothesise that sex is a major factor driving differentiation patterns, namely, the distribution of genomic signatures within autosomes (gene expression and alternative splicing) and sex chromosomes (fixed SNPs). In addition, we hypothesise that a full transcriptomic analysis will bring forth novel relationships on the sex‐colouration axis, thus expanding and/or complementing the previous gene‐targeted approach.

## 2 Material and Methods

### 2.1 Phenotyping, RNA Extraction and Sequencing

The data for this work was collected from juvenile owls that were taken from the wild at about 25 days post‐hatching (when they are still in the nest) and kept in captivity in single‐bird aviaries in Lund, Sweden. Feather bud tissue was collected from 32 juveniles 50 days after hatching (a period chosen after acclimatization to aviary conditions) and stored at −80°C (all information about individual specimens can be found in Table S1). Individuals were phenotyped for sex and colour morph according to Karell et al. (2011), since those traits are already noticeable at 50 days after hatching. Weight was measured at two time points: 15 days post‐hatching and then again at 90 days post‐hatching. We then performed *t*‐tests considering sex as the explanatory variable. Feather bud tissue was sent to BGI, China, for RNA extraction and sequencing, which was performed with DNBSEQ chemistry and the strand‐specific library preparation protocol able to produce 100 bp paired‐end reads.

### 2.2 Transcriptome Assembly, Annotation and Gene Models

Transcriptomic data was utilized to build a coherent genome annotation for downstream use in gene expression analysis. RNA‐seq reads were processed with the nf‐core/genomeannotator pipeline (github.com/marchoeppner/genomeannotator) version 1.0dev. The workflow combines different types of annotation evidence and synthesizes them into a consensus gene build using a range of established tools (https://github.com/marchoeppner/genomeannotator/blob/dev/CITATIONS.md). Included in this process were both species‐specific RNA‐seq data as well as gene models from the burrowing owl (*Athena cunicularia*) and the chicken ( *Gallus gallus* ) from EnsEMBL release 110 that we syntenically projected onto our assemblies. We utilized previously published tawny owl genome (Baltazar‐Soares et al. 2024) as a template for gene model building, which was assembled from a tawny owl male. In this workflow, different types of evidence are processed and aligned against a repeat‐masked assembly to inform the computation of a consensus gene build. We further utilized protein sequences from Uniprot—limited to birds and with support from either protein and/or transcriptome data—synthetically mapped gene models from the bird species previously mentioned and transcriptome data generated for this study. The completeness of the resulting gene builds was estimated using BUSCO (version 5.3, PMID 34320186) against the aves_odb10 reference database. To functionally annotate each respective gene build, the final GFF3 file was analysed with EggnogMapper (version 2.1.7, PMID 34597405) against the core database (downloaded on 2022‐04‐25).

### 2.3 Alignment, Mapping and Read Quantification

In order to proceed with differential expression analyses, RNA‐seq reads were aligned against the tawny owl genome with HISAT2 v2.1.1 (Kim et al. 2019) considering strandness and reverse orientation of the forward read. All other parameters were maintained as default. Aligned files were sorted and indexed with Samtools v 1.16.1 (Li et al. 2009) and subsequently utilized to sort and index bam files. Reads were counted with HTSeq v2.0.3 (Anders et al. 2015) with the non‐stranded option for paired‐end data.

### 2.4 Differential Gene Expression Analyses

HTSeq read counts were filtered for a minimum count sum of one per gene and per sample in the R statistical environment v4.3.1 (R Core 2013). Raw read counts were normalized using the standard analysis pipeline of the package *DESeq2* v1.42 (Love et al. 2014). Normalized read counts were quality‐checked through dispersion estimate plots and samples were checked for outliers using a sample clustering approach on regularized log‐transformed expression. All samples passed the quality check and were used to test for the effect of sex, morph colouration and their interaction on gene expression. Normalized reads were plotted using *ggplot2* v3.4.4 (Wilkinson 2011) and *pheatmap* v1.0.12 (Kolde and Kolde 2015). Gene ontology (GO) term enrichment within the differentially expressed genes was assessed using the *GOstats* v2.68 (Falcon and Gentleman 2007) and *GSEABase* v1.64 packages (Morgan et al. 2019) and corrected for multiple testing using the false discovery rate method implemented in *goEnrichment* v1.0 (Hallab 2015).

### 2.5 Alternative Exon Usage

Differential exon usage, which is indicative of alternative splicing, was analysed using DEXSeq v1.48 (Anders et al. 2012). Therefore, we first prepared the annotation file running the dexseq_prepare_annotation.py script, which is part of the DEXSeq pipeline. Afterwards, HISAT2 aligned reads were counted for each exon using *dexseq_count.py* with the parameters ‐s no ‐p yes. In the R v4.3.1 statistical environment, we calculated differential exon usage by contrasting the full model (~ sample + exon + Factor 1: exon + Factor 2: exon) with the reduced model (~ sample + exon + Factor 1: exon) to get the effect of Factor 2 while accounting for Factor 1 as covariable. Thereby, we tested the Sex effect (Sex as Factor 2 and Morph as Factor 1) and the Morph effect (Morph as Factor 2 and Sex as Factor 1) separately.

### 2.6 Variant Calling and Outlier Analyses

In order to identify SNP outliers associated with colour or sex at the transcriptomic level, we performed variant calling on individual‐based (non‐pooled) transcriptomes and submitted them to *F*_ST_—based outlier detection tests. Variant calling was performed with GATK's v4.5.0.0 (Van der Auwera and O'Connor 2020) pipeline according to *GATK's best practices* for *RNAseq short variant discovery* (SNPs + Indels) (DePristo et al. 2011). Hard‐filtering steps were customized with the following parameter definition after testing for sensitivity in variant removal: QD < 2.0; QUAL < 30.0; SOR > 2.0; FS > 60.0; MQ < 60.0. Lastly, we utilized bcftool*s* v.1.18 (Danecek et al. 2021) to extract only those bi‐allelic variants with minor allelic frequency > 0.05, and missing rate below 30% that were mapped on coding regions of the tawny owl genome. In order to investigate whether transcript‐derived SNPs were fixed between groups, that is, colour and sex, we performed *F*_ST_—based outlier detection with the Bayesian framework implemented in Bayescan v 2.1 (Foll and Gaggiotti 2008). We utilized the following set of general parameters: 20 pilot runs with a length of 5000 chains and a burn‐in length of 100,000 while testing for a prior of 100 and a false discovery rate of 0.05. Lastly, we also performed gene ontology (GO) terms enrichment analyses to explore whether biological, metabolic or cellular processes could be significantly represented among genes where outliers have been detected in Panther 1.80 GO Ontology database (DOI: 10.5281/zenodo.1053640).

## 3 Results

### 3.1 Body Weight at Early Stages Reflects Onset of Sexual Dimorphism in Tawny Owls

Exploring body size dimorphism between sexes revealed that weight (measured both at 15 days post hatching and at 90 days post hatching) is already different between sexes at early stages (*t*‐test, females × males = 2.278, *p* = 0.03) and final 90 dph (*t*‐test, females × males = 6.32, *p* < 0.001), though weight gain and weight increased over the 75 day time period was not shown to significantly differ (*p* = 0.06 and *p* = 0.23 for weight gain absolute and weight gain %, respectively) (Figure S1).

### 3.2 Differential Gene Expression Indicates Male Specific Expression and no Dosage Compensation

**FIGURE 1:**
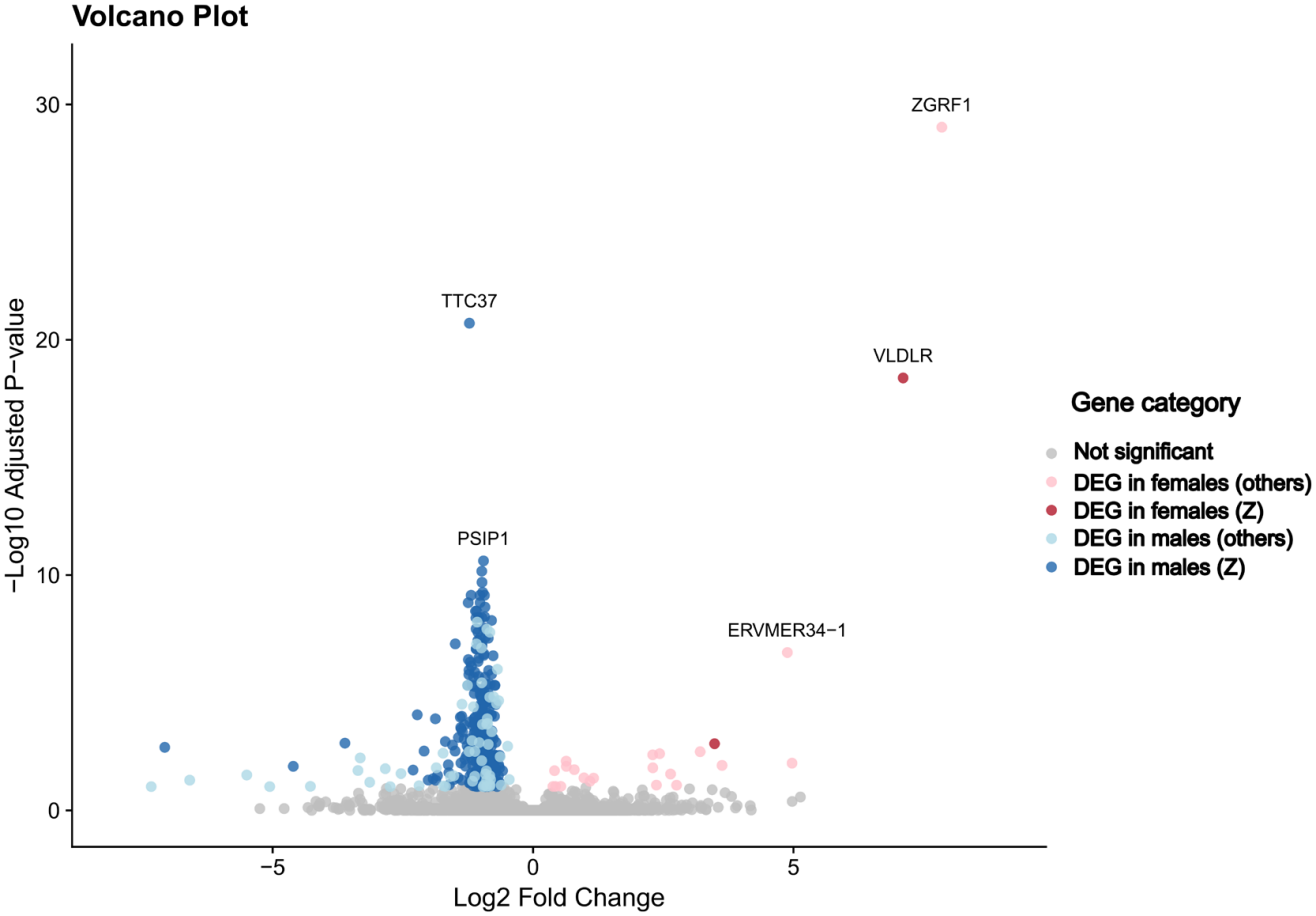
Vulcano Plot of differentially expressed genes between the sexes. Each point represents one gene showing its respective log2 fold change (*x*‐axis) and −log10 adjusted *p*‐value (*y*‐axis). Differentially expressed genes in males and females are indicated in blue and red respectively and distinguishing whether mapped to Z chromosome or other (autosome or unmapped). Not significantly differently expressed genes are indicated in grey. Gene names are plotted for five highly significant genes.

**FIGURE 2:**
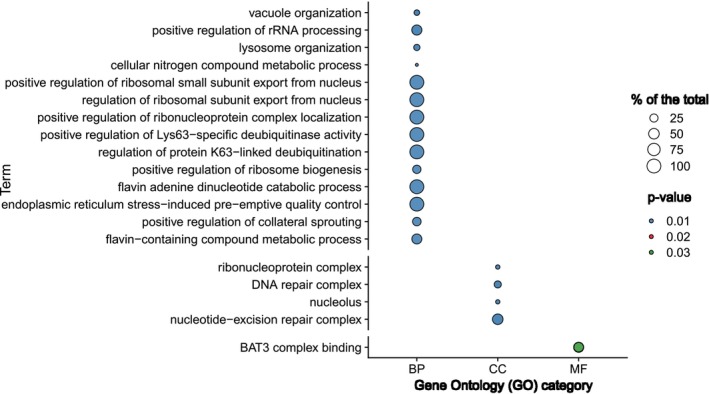
Enriched gene ontology terms within the sex DEGs. Categories on the *x*‐axis include biological processes (BP), cellular components (CC) and molecular functions (MF). The percentage of genes within a GO term that are among the DEGs is displayed as circle size, and the colouration reflects the FDR corrected *p*‐value.

Transcriptome sequencing was performed for 32 individuals. Reference gene build completeness yielded a combined gene space coverage (complete and fragmented models) of 95.7%. On average, 46 million reads (SD: +/−1.8 million) were aligned per individual sample against the tawny owl genome with an average mapping rate of 72.8% (SD: +/−3.81%) (Table S2). We tested for the effect of sex, morph, and their interaction on gene expression in developing feather buds. We identified a total of 352 differentially expressed genes (DEGs) between the sexes (two in interaction with morph) from our dataset (*p*‐value < 0.1). Sex comparisons further showed that most sex DEGs were related to males, which certainly is impacted by males being the homogametic sex in birds (Figure 1). To understand whether the link between the increased expression in males and the location of these genes in the genome, we mapped them to the chicken genome and classified the sex DEGs into three groups: genes that mapped on the chicken Z chromosome (*n* = 272; 2 in females, *VLDLR* and *CCDC171* and 270 in males), genes mapped outside of the chicken Z chromosome (*n* = 34; 15 upregulated in females and 19 upregulated in males) and unmapped genes (*n* = 46; 6 in females and 40 in males) (Figure S2, Table S3 and Table S4). Since we cannot rule out that the most parsimonious explanation for the 270 Z‐linked genes in males is showing a higher expression compared to females being due to lack of complete dosage compensation, we abstain from referring to upregulation in those cases. The functions of these genes are related to enrichment for ribosomal regulation and activity (Figure 2).

**FIGURE 3:**
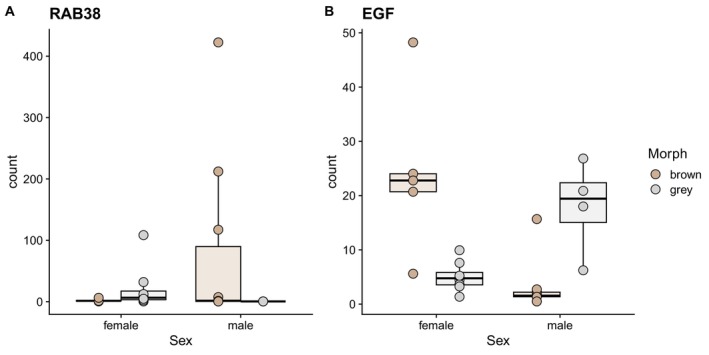
Read counts for RAB38 and EGF, the two genes showing a significant interaction between morph and sex. DESeq2 was used to normalize gene counts (*y*‐axis). Colours indicate the morph type.

Contrary to our expectations, we found no differentially expressed genes (DEGs) between the colour morphs and only two genes showed an interaction between morph and sex. One of them is a *RAS* oncogene family member, *RAB38* and the other one is related to the growth factor EGF (Figure 3).

Analysing pathway enrichment in sex‐specific differentially expressed genes revealed that biological functions related to nucleotide‐excision repair complex (GO:0000109), DNA repair complex (GO:1990391), melanosome synthesis (GO:0042470) and pigment granule (GO:0048770) with > 10× fold enrichment and significant FDR < 0.05 (Table S5).

### 3.3 Sex and Colour Morph Suggests the Use of Different Exons

We identified one gene with differential exon usage between the sexes, *SLC12A*. Three isoforms have been reported for this gene (‘UniProt: the universal protein knowledgebase in 2023’, 2023). Females showed a reduced read count on exon 12, suggesting the possibility that this exon gets spliced out. A comparison between morphs resulted in three genes with differential exon usage: *CD151* (10 reported isoforms), *DKC1* (2 reported isoforms) and *EPS8L2* (3 reported isoforms) with lower read counts in the grey morph in exon 7 (last exon), exon 10 and exon 13, respectively (‘UniProt: the universal protein knowledgebase in 2023’, 2023) (Figure S4, Table S6).

### 3.4 Transcript Single Nucleotide Polymorphisms Suggest Colour and Sex‐Specific Variation

**FIGURE 4:**
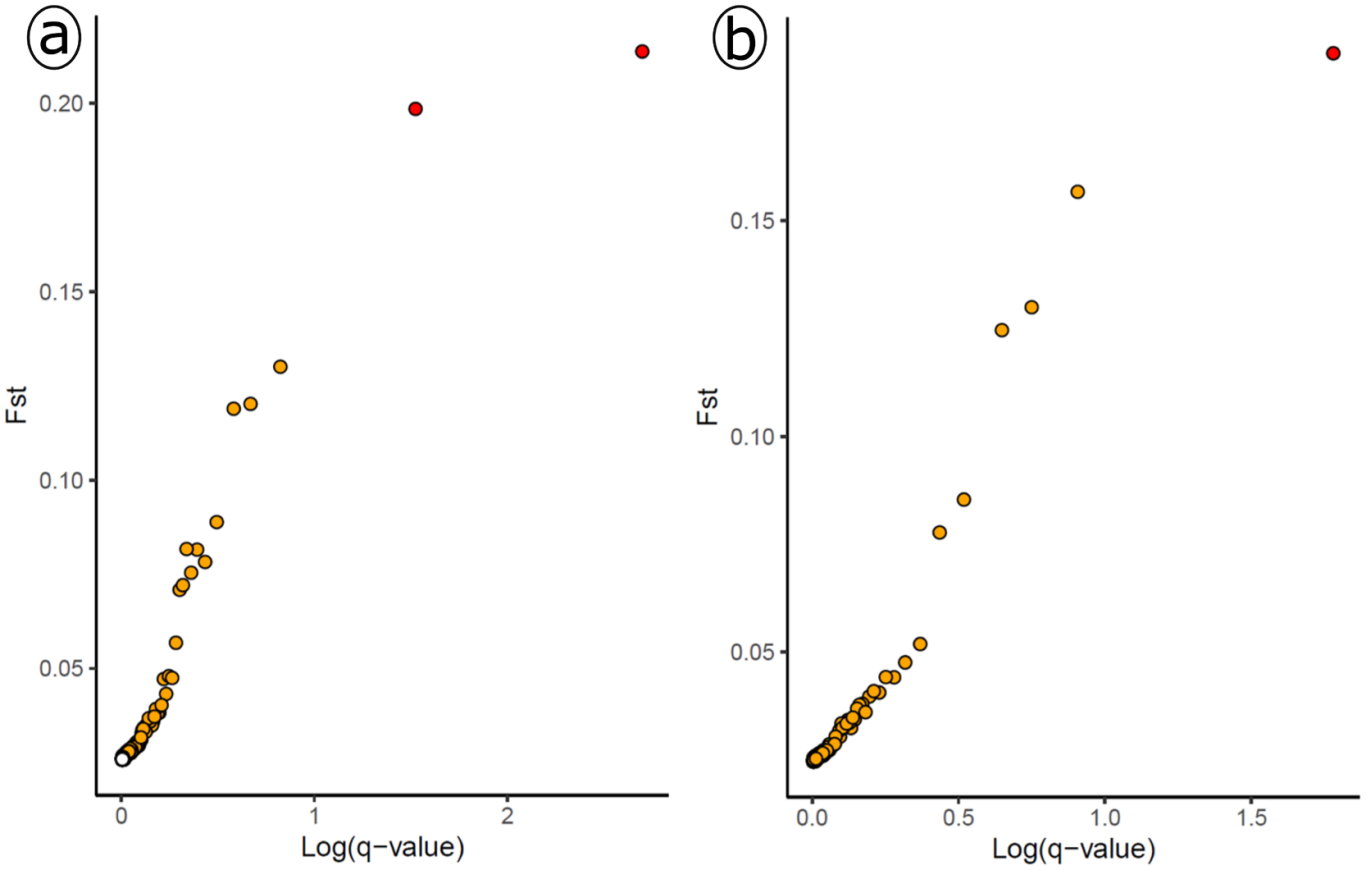
Outlier detection analyses for sex (a) and colour morph (b). The *x*‐axis shows the log (*q*‐value), the *y*‐axis represents the *F*_ST_ pairwise comparison between groups. The loci identified under directional selection are shown in red, while orange represents neutrality (for prior odds = 100).

A total of 65,222 SNPs were retained for final analyses (see filtering steps in Table S7). Outlier detection analyses revealed 2 and 1 outliers for sex and colour morph, respectively. For sex, outliers were in genes *GHR* (*F*_ST_ = 0.21, *q*‐value = 0.002) and *GSK3B* (*F*_ST_ = 0.20, *q*‐value = 0.03), located on chicken chromosomes Z and 5. The single outlier detected for colour morph was in the gene *C2CD5* (*F*_ST_ = 0.18, *q*‐value = 0.001), located in chicken's chromosome 1 (Figure 4) (Tables S8, S9 and S10).

## 4 Discussion

Intraspecific comparative transcriptomics allows us to understand microevolutionary processes at finer scales, and to explore phenotype diversity when the genomic background is shared. According to expectations, screening the transcriptomes of juvenile tawny owls revealed sex‐specific expression patterns with emphasis on genes located in the sex chromosome.

### 4.1 Sexually Dimorphic Gene Expression Patterns Suggest a Superimposition on Underlying Sex‐Specific Genomic Variation

Species exhibiting sexual dimorphism have to overcome the conflict originated by opposite selective pressures acting on each sex to produce divergent phenotypes, while the shared genomic background drives phenotypes to be equal across sexes (Roff and Fairbairn 2007). In our study, we observed that inter‐sexual variation in terms of differentially expressed genes was detected at a much higher magnitude than genomic variation in the same areas (DEGs = 350 vs. SNPs = 2), though our SNP discovery strategy did not encompass the screening of dosage compensation mechanisms. In birds, sex‐specific expression differences have been reported from early embryogenesis towards the development and maturation of gonads (Mank et al. 2010; Tagirov and Rutkowska 2014). However, we believe our results offer complementary information to what we know on sex‐specific gene expression and particularly their association with sexual dimorphism. Noteworthy, our genome was assembled from a male tawny owl, and thus we did not possess information on the W chromosome. This means that our results should only be interpreted in the light of Z chromosome and that we still have substantial knowledge gaps on the contribution of W in the tawny owl system. The evolution of W chromosome is nevertheless characterized by a high level of degeneracy and respective gene loss (Huang et al. 2023). As a reference, a total of 38 homologous pairs were reported between Z‐W chicken chromosome pairs (Huang et al. 2023) from a total of approximately 80 protein‐coding genes in the most recent W build (Warren et al. 2017). The W chromosome is also evolutionarily conserved (Smith et al. 2023), as none of female‐specific DEGs had a known homologous in the Z chromosome of the chicken.

Because we were dealing with live specimens, we were unable to sample traditional sex determination‐linked tissues, like gonads or brain, which is where most of the sex‐specific physiological (hormones) and molecular (gene expression) patterns have been reported (Friedrich et al. 2022; Naurin et al. 2011). By identifying sex‐specific DEGs on an organ (tegument) that, to the best of our knowledge, is not directly involved in sex determination nor in gonadal development, our results might be revealing the expected but perhaps seldom investigated cascading effects of a resolved sexual conflict (absence of conflict), which overall reflects sex‐specific selection pressures later in the life cycle (Mank et al. 2010).

### 4.2 Genome Location and Function of Both DEGs and Gene‐Bearing SNPs Are Suggestive of Sex‐Specific Phenotypes

The large majority of the DEGs (272/352) were found to be located within the putative Z chromosome of the owl. Complete dosage compensation (1:1 expression ratio between sexes) as it exists in mammals due to a second copy of Z chromosome in the homogametic sex would nevertheless be unexpected in a bird, since partial compensation mechanisms (which can be taken the form of gene‐specific or regional gene shutdown) are known to exist in chicken, crows and collared flycatchers (Graves 2014; McQueen et al. 2001). Though differential expression on the Z chromosome suggests non‐compensation of the target gene product, implications for the organismal function are beyond the scope of this study. Overall, GO term analyses revealed higher expression of a wide breadth of biological processes, mostly linked to ribosomal activity and developmental processes. The most significantly expressed genes varied in putative downstream functions. For example, *TTC37*, which we found to be differentially expressed in male tawny owls, was reported to be a non‐compensated Z‐chromosome gene involved in chicken's sexual determination machinery (Soler et al. 2021); *PSIP1*, also differentially expressed in male tawny owls, was shown to be a key regulator of transcription in zebra finches (Luo et al. 2012). As for females, the upregulated gene *ZGRF1* is a relatively unknown zinc‐finger with a single report outside the medical context related to high intramuscular fat deposition in bovines (Poleti et al. 2018), and *VLDLR* was identified as a potential modulator of egg production and performance in poultry (Han et al. 2009; Wang et al. 2016). Importantly, the two genes upregulated in females are found to be located in Z chromosome, *VLDLR* and *CCDC171*, suggesting these could be overcoming the standard (partial) dosage compensation that exists on the avian Z (Graves 2014).

Lastly, the two SNPs identified as outliers when factoring for sex are located in genes *GHR* and *GSK3B*, that are known modulators of growth and behaviour respectively (Harvey 2013; Kaidanovich‐Beilin and Woodgett 2011; Lee et al. 2007). Resuming, the observation that the effects of differentially expressed genes (growth, behaviour, egg production) relate to either known sexually divergent phenotypes (females are larger than males, males are more exploratory) or sex‐specific phenotypes (egg laying) grants some validation to our findings.

### 4.3 Male‐Specific Colouration Pathway Enrichment and Key Melanogenesis Gene Upregulated in Brown Males Suggest Sex‐Specific Timing of Colouration

We found no differentially expressed genes when factoring for colour morph, despite previous studies reporting otherwise in tawny owls (Emaresi et al. 2013). A possible explanation is that our sampling strategy missed the timing during which the machinery orchestrating pigment formation, migration and deposition is active. Our samples were taken at 50 days post‐hatching, while Emaresi and colleagues worked with two time points, 11‐ and 25‐days post‐hatching. Another explanation is that the tissue we utilized (feather buds, as opposed to blood in Emaresi et al. 2013) was not optimal to identify gene expression of the melanocortin pathway (active in the hypothalamus) and/or melanin production pathway (in melanocytes). However, we detected two lines of evidence suggesting the timing of colouration to be sex‐specific. The first is 10× fold enrichment for melanosome—a cytoplasmic organelle within which melanin pigments are synthesized and stored—and granule pigmentation—membrane‐bounded vesicle containing pigment and/or pigment precursor molecules—pathways led by upregulation of Tyrosine (*TYR*), protein melan‐A (*MALENA*) and Tyrosinase‐related protein 1 (*TYRP1*). Tyrosine is the precursor molecule of melanin (Rzepka et al. 2016), whose oxidation (by tyrosinase) is a critical step in melanin biogenesis (Riley 1997); the second is the upregulation of autosomal genes involved in melanogenesis (*RAB38*) and epidermis development (*EGF*) in covariation with sex (Kim et al. 2020; Xi et al. 2020). Brown females exhibited an upregulation of *EGF*, while brown males showed an upregulation of *RAB38*, which is active during melanogenesis. In the context of the tawny owls' body‐size sexual dimorphism, our results might suggest sex‐specific timing of melanogenesis and putative epidermal structures mediated by *EGF*.

### 4.4 Sex‐ and Colour Morph‐Specific Differential Exon Usage Suggest Alternative Phenotype Production Mechanism

Transcripts produced by alternative splicing confer another layer of molecular variation on top of differential gene expression (S. Anders et al. 2012). This is because exons with beneficial/maladaptive effects specific to an intraspecific group, such as sex or colour morph in our case, can be selected for expression/spliced out if present in the right/wrong group (Rogers et al. 2021). Here we only detected one gene, *SLC12A*, with sex‐specific exon usage. The gene codes for a cross‐membrane solute carrier of the 12A superfamily that has been shown to modulate chloride‐coupled cations across membranes, as well as behaviour in multiple species (Trejo et al. 2023), but we were unable to find any information that could link our results with sexual function or sex‐specific phenotypes. Alternative exon usage between colour morphs was detected in three genes, *CD151*, which codes for an antigen, *DKC1*, a ribonuclear protein and *EPS8L2*, yet another gene coding for an epidermal growth factor receptor. While the fact that these genes possess known isoforms in humans might help to validate findings of alternative exon usage in other species, evidence of functionality remains elusive.

## 5 Conclusions and Future Directions

Sexually dimorphic species offer unique opportunities to understand the link between intraspecific molecular variation and functional, yet sex‐specific, phenotypes. While in our study we were unable to sample key organs or follow the specimens until later life stages, we provide multiple levels of molecular evidence—differential expression, single nucleotide polymorphisms and alternative exon usage—that all point towards a resolved sexual conflict affecting growth‐related differences between the sexes. The interaction between sex and colour is relevant in the context of our previous work, which has suggested two SNPs linked to colour polymorphism (Baltazar‐Soares et al. 2024) also Figure S4. We found those genes expressed but no significant interaction between morphs. In general, we believe our study exemplifies how multiple analytical approaches to molecular data might help to overcome limitations associated with working with non‐model, wildlife organisms, such as the capacity to maintain subjects in captivity during critical developmental stages, or the sampling of vital organs that are hotspots of transcriptomic activity. However, we fully acknowledge that future studies should be able to explore technical aspects that were unable to do so in this work. For example, disentangling lack of dosage compensation from up‐regulation in *Z*‐linked male individuals is essential to validate not only expression patterns but also identify putative cis‐regulation that could exist in non‐transcribed regions. In addition, performing qPCR in candidate genes at key developmental times would further reinforce the biological significance of the findings.

## Author Contributions

All authors contributed to the design of the research. M.B.‐S. performed the research; M.B.‐S., M.J.H. and M.P.H. analysed the data. J.E.B. contributed with analytical tools. M.B.‐S. wrote the paper and all authors contributed to its final form.

## Funding

The work developed in this manuscript was supported by the Academy of Finland funding decision 321417 attributed to J.E.B. and decisions 309992, 314108 and 335335 attributed to P.K.

## Disclosure

Benefits Generated: A research collaboration was developed with scientists from the countries providing genetic samples; all collaborators are included as coauthors, the results of research (and similar research on the topic by our group) have been shared with the broader scientific community and with the public. Our group is committed to international scientific partnerships, as well as institutional capacity building.

## Conflicts of Interest

The authors declare no conflicts of interest.

## Supporting information


**Figure S1:** Aviary weight measurements. Weight has been measured in two time points, 15 days post hatching and 90 days post hatching. Here we present pairwise comparisons between sexes. Note that only significant interactions (*t*‐test, *p* < 0.05) are depicted in the picture. *p = 0.05, ***p < 0.01.

**Figure S2:** MA plot for differentially expressed genes between sexes. Distribution of log‐fold changes across expressed genes. In grey, expressed genes that do not significantly differed in expression levels. In blue, differentially significantl expressed genes (positive *y*‐axis for females and negative *y*‐axis for males).

**Figure S3:** DEGs among different groups: Heatmap showing 352 differentially expressed genes between the sexes. Samples are organized through a hierarchical clustering approach and cluster well into females and males for the expression of the sex DEGs. Grey and brown colours on top of the heatmap indicate the morph type and purple colours on the left side of the plot distinguish the two gene groups clustered by similarity in expression. Blue colours group the genes by location into *Z* chromosome located, not *Z* chromosome located and not mapped to chicken genome. *Z* scores for expression differentiation are coloured along a blue—red colour gradient.

**Figure S4:** Differential exon usage: Read counts per exon are plotted to visualize differential exon usage in one gene linked with sex and three genes linked with morph. For each gene, we plotted expression (fitted count estimated from the glm regression), exon usage (fitted count estimates standardized for gene expression average to visualize the exon usage effect only) and the gene (exons in blocks, introns as lines) along the genomic region of the respective gene. Differentially used exons are indicated in purple and colours indicate the morph and sex types.

**Figure S5:** Network of GOterms identified among differentially expressed genes in males.

**Table S1:** Information on individual specimens utilized in this work.
**Table S2:** Statistics of transcripts mapping to reference with HISAT2.
**Table S3:** Differentially expressed genes identified in this study in relation to location of orthologues in the chicken genome version (GRCg7b).
**Table S4:** All differentially expressed genes and respective statistics. Genes were retained for a *p*‐value (adj) < 0.1.
**Table S5:** Enriched pathways on differentially expressed genes on males, ranked by a combination of FDR (cut off > 0.05) and fold enrichment.
**Table S6:** Alternative exon usage identified between sexes and colour morphs.
**Table S7:** Number of SNPs retained after each filter step.
**Table S8:** SNPs outliers (*F*_ST_) when factoring for sex. The * denotes significant results for prior odds = 100.
**Table S9:** SNPs outliers (*F*_ST_) when factoring for colour morph. The * denotes significant results for prior odds = 100.
**Table S10:** Enriched pathways of all genes found to co‐vary with colour morph—from this work and from Baltazar‐Soares et al. 2024 (^+^). Ranked by a combination of FDR (cut off > 0.05) and fold enrichment.

## Data Availability

Scripts utilized in this work can be found in Github: https://github.com/Miguel‐BSoares/tawny_owls_genomic_resources; github.com/M‐Heckwolf; github.com/marchoeppner/. Mapped RNA‐seq reads are deposited as *fastq* files in the BioProject PRJNA988428 in the SRA and released upon publication. A new version of the genome will also become publicly available in the same repository.
