# Supplementary figures and images for: Transcriptomics Reveal Molecular Signatures of a Resolved Sexual Conflict and Potential Association With Colour Polymorphism in Tawny Owls
Source: Mol Ecol. 2026 Apr 10;35(7):e70338. doi: 10.1111/mec.70338 (PMC13069001; doi:10.1111/mec.70338)

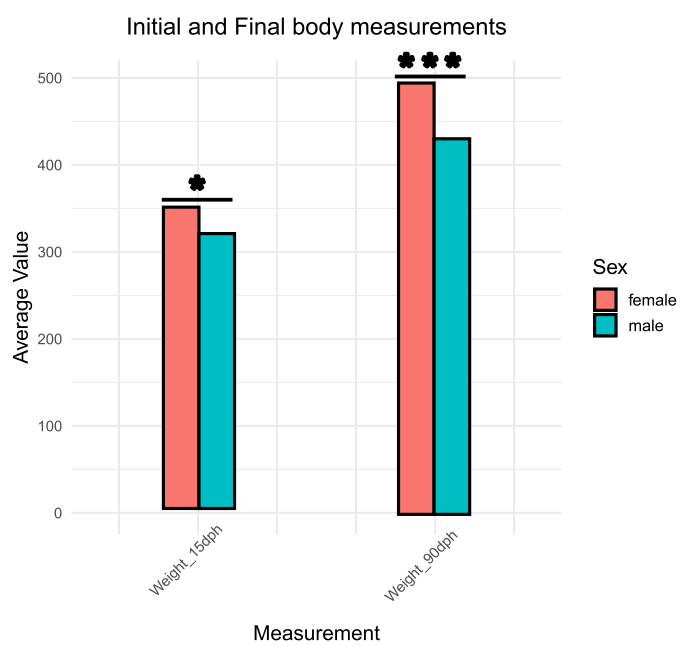

Supplement: Supplementary file 1 — Figure S1: Aviary weight measurements. Weight has been measured in two time points, 15 days post hatching and 90 days post hatching. Here we present pairwise comparisons between sexes. Note that only significant interactions (t‐test, p < 0.05) are depicted in the picture. *p = 0.05, ***p < 0.01. [file MEC-35-e70338-s004.pdf]

MA Plot Sex LQ

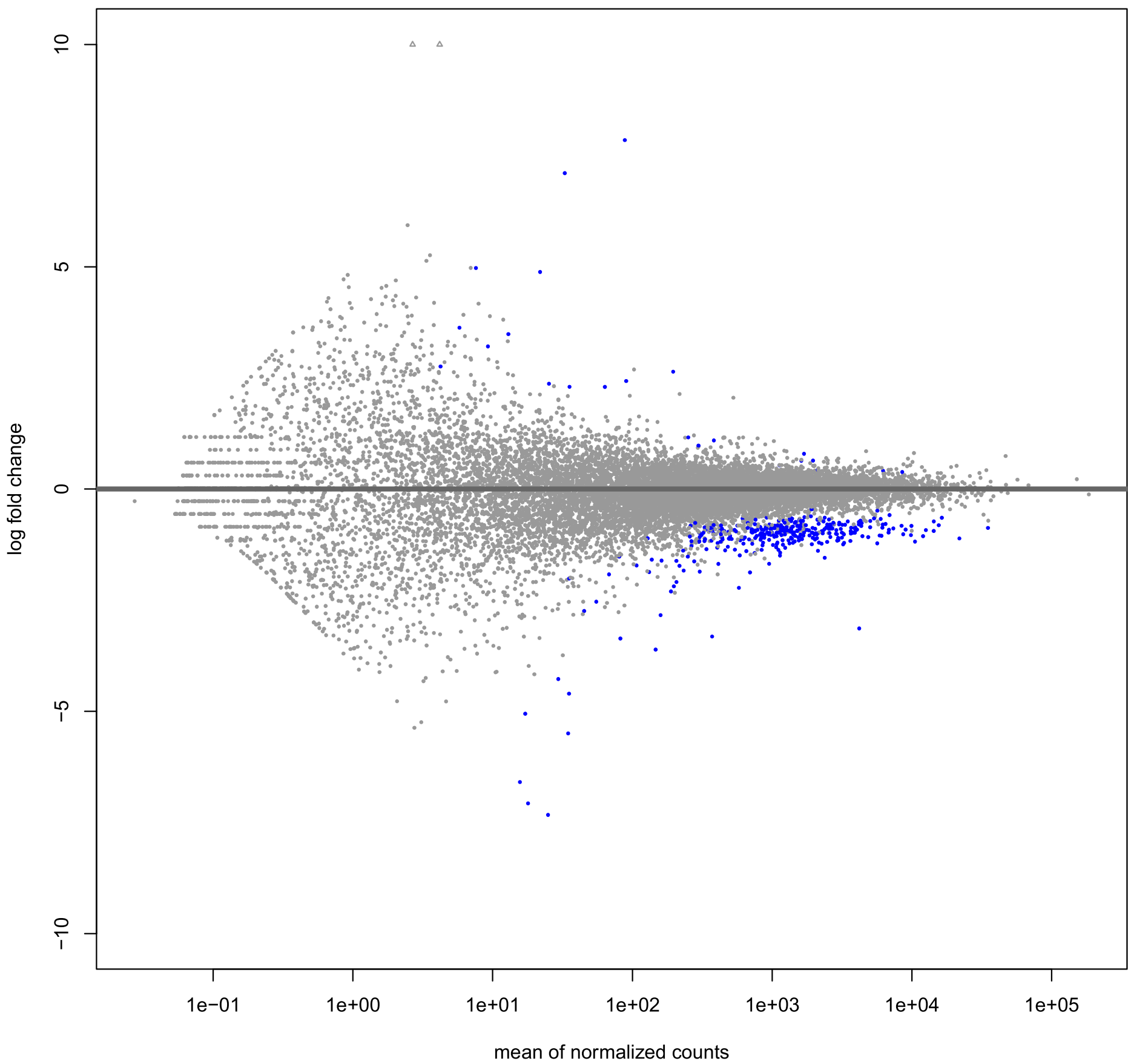

Supplement: Supplementary file 2 — Figure S2: MA plot for differentially expressed genes between sexes. Distribution of log‐fold changes across expressed genes. In grey, expressed genes that do not significantly differed in expression levels. In blue, differentially significantl expressed genes (positive y‐axis for females and negative y‐axis for males). [file MEC-35-e70338-s003.pdf]

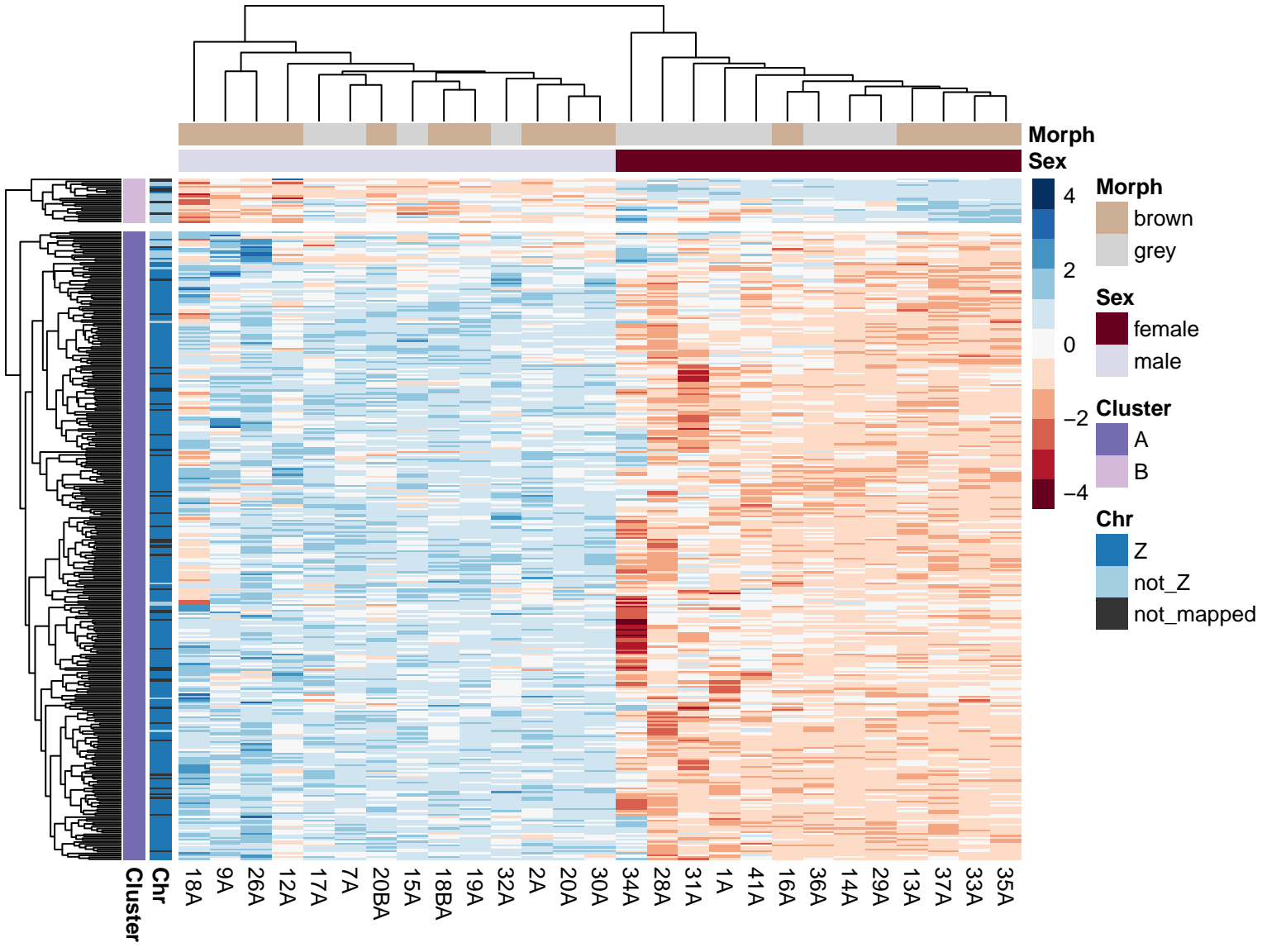

Supplement: Supplementary file 3 — Figure S3: DEGs among different groups: Heatmap showing 352 differentially expressed genes between the sexes. Samples are organized through a hierarchical clustering approach and cluster well into females and males for the expression of the sex DEGs. Grey and brown colours on top of the heatmap indicate the morph type and purple colours on the left side of the plot distinguish the two gene groups clustered by similarity in expression. Blue colours group the genes by location into Z chromosome located, not Z chromosome located and not mapped to chicken genome. Z scores for expression differentiation are coloured along a blue—red colour gradient. [file MEC-35-e70338-s001.pdf]

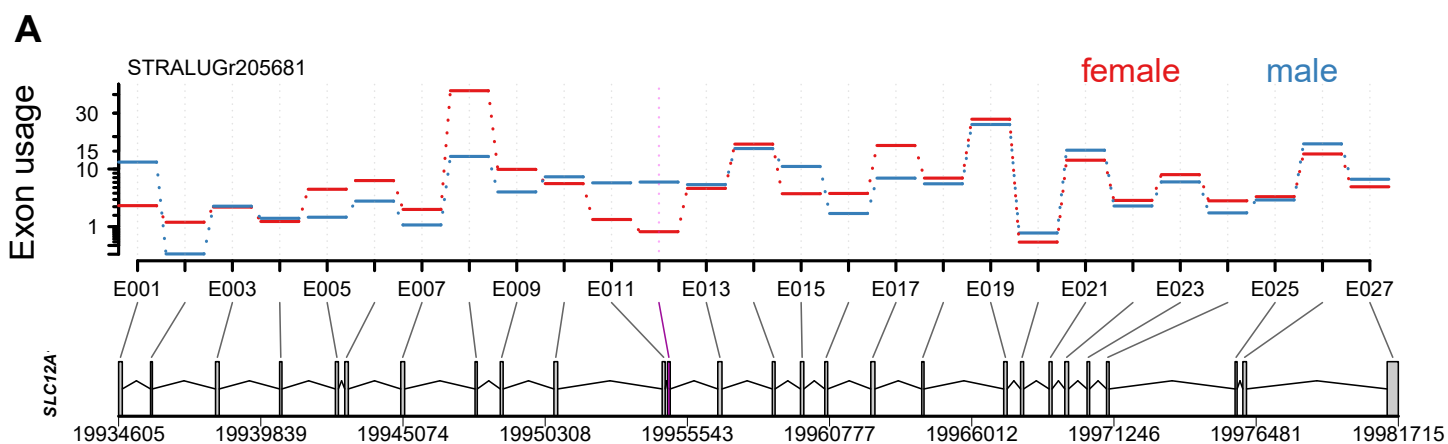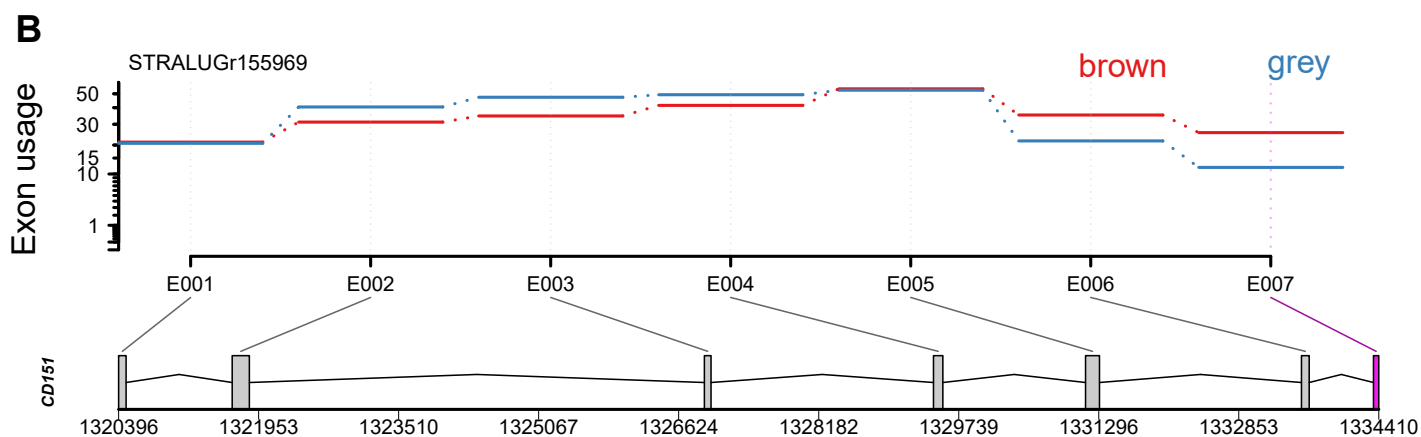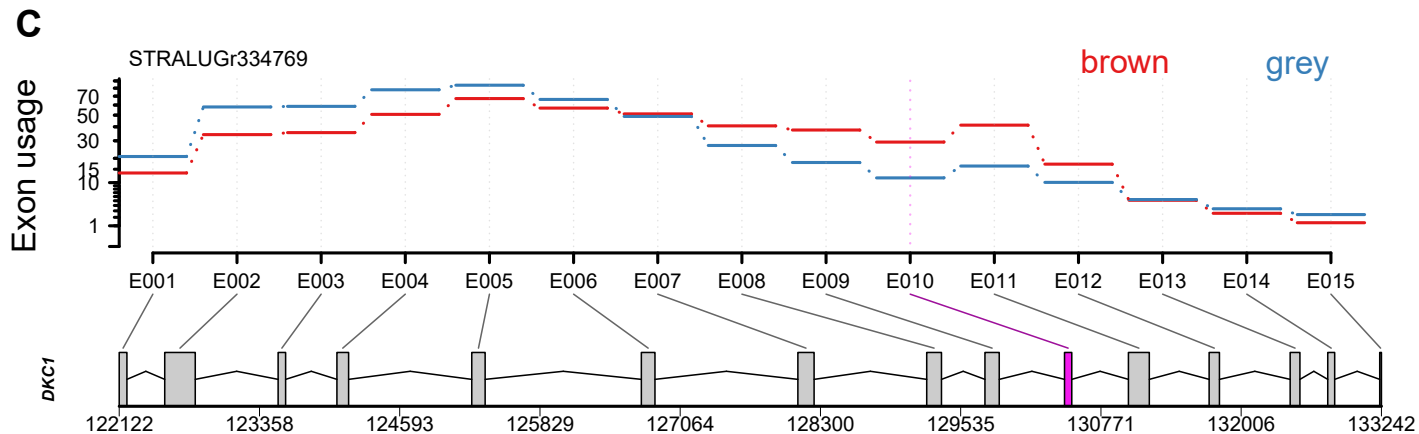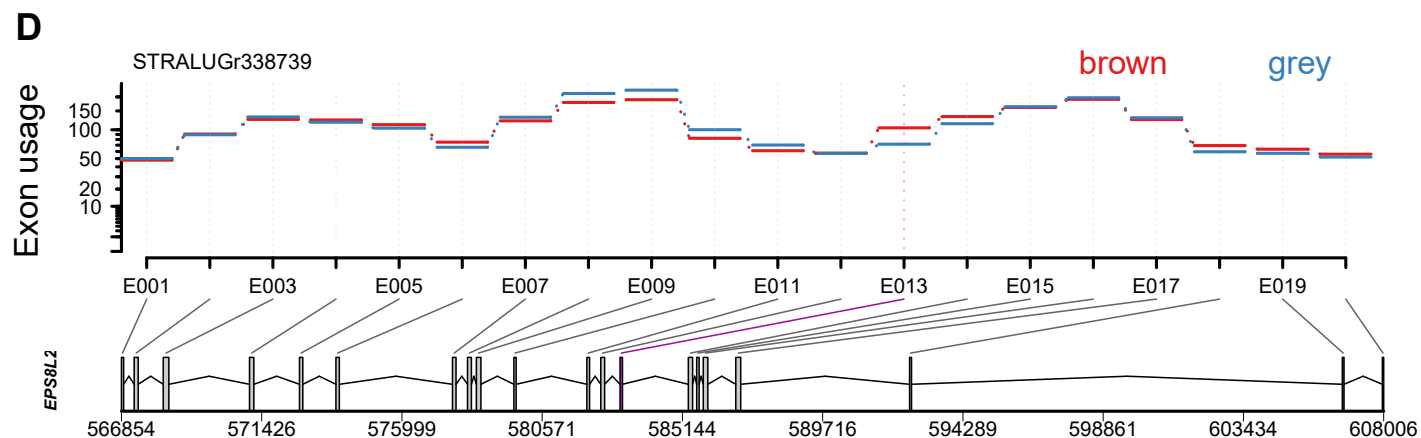

Supplement: Supplementary file 4 — Figure S4: Differential exon usage: Read counts per exon are plotted to visualize differential exon usage in one gene linked with sex and three genes linked with morph. For each gene, we plotted expression (fitted count estimated from the glm regression), exon usage (fitted count estimates standardized for gene expression average to visualize the exon usage effect only) and the gene (exons in blocks, introns as lines) along the genomic region of the respective gene. Differentially used exons are indicated in purple and colours indicate the morph and sex types. [file MEC-35-e70338-s002.pdf]

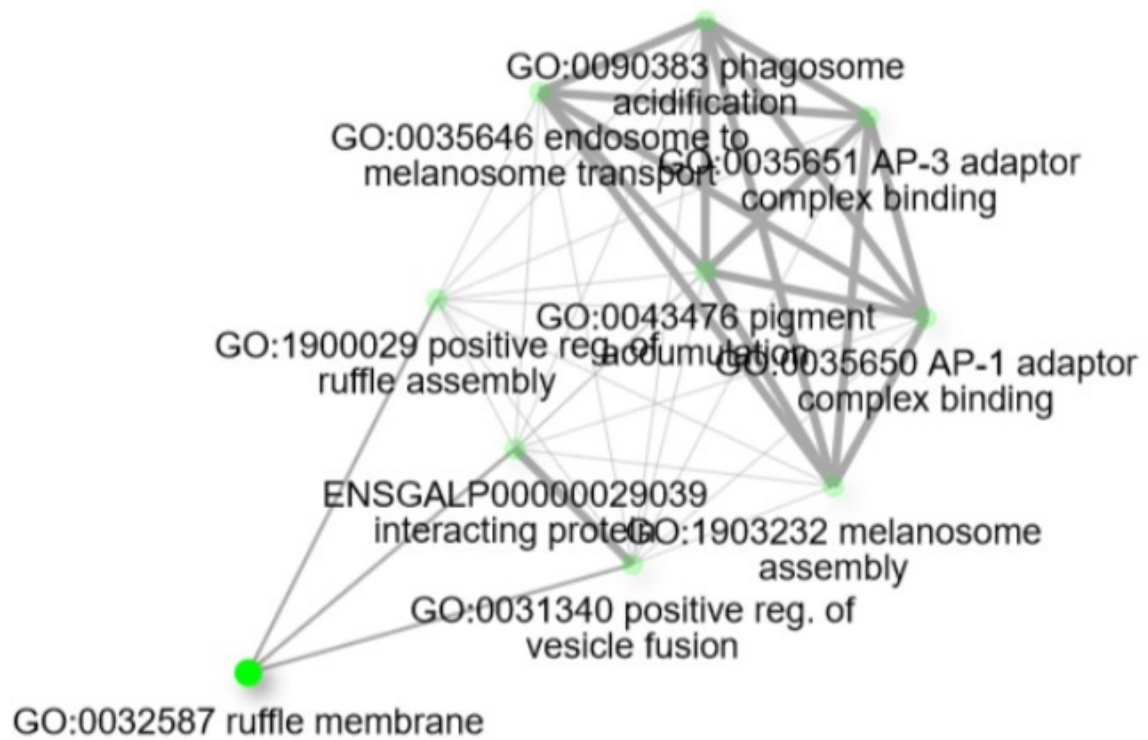

Supplement: Supplementary file 5 — Figure S5: Network of GOterms identified among differentially expressed genes in males. [file MEC-35-e70338-s005.pdf]
