# Supplementary material for: Transcriptomics Reveal Molecular Signatures of a Resolved Sexual Conflict and Potential Association With Colour Polymorphism in Tawny Owls
Source: Mol Ecol. 2026 Apr 10;35(7):e70338. doi: 10.1111/mec.70338 (PMC13069001; doi:10.1111/mec.70338)
Supplement: Supplementary file 6 — Table S1: Information on individual specimens utilized in this work. Table S2: Statistics of transcripts mapping to reference with HISAT2. Table S3: Differentially expressed genes identified in this study in relation to location of orthologues in the chicken genome version (GRCg7b). Table S4: All differentially expressed genes and respective statistics. Genes were retained for a p‐value (adj) < 0.1. Table S5: Enriched pathways on differentially expressed genes on males, ranked by a combination of FDR (cut off > 0.05) and fold enrichment. Table S6: Alternative exon usage identified between sexes and colour morphs. Table S7: Number of SNPs retained after each filter step. Table S8: SNPs outliers (F ST) when factoring for sex. The * denotes significant results for prior odds = 100. Table S9: SNPs outliers (F ST) when factoring for colour morph. The * denotes significant results for prior odds = 100. Table S10: Enriched pathways of all genes found to co‐vary with colour morph—from this work and from Baltazar‐Soares et al. 2024 (+). Ranked by a combination of FDR (cut off > 0.05) and fold enrichment. [file MEC-35-e70338-s006.docx]

**Supplemental Information for:**

**Transcriptomics reveal molecular signatures of a resolved sexual conflict and respective association with colour polymorphism in tawny owls**

Miguel Baltazar-Soares^1^, Melanie J. Heckwolf^2^, Marc P. Hoeppner^3^, Patrik Karell ^4,5,6^, Dominic Wright^7^, Jan-Åke Nilsson^4^, Jon E. Brommer^1^

**Table of Contents:**

| **Supplemental tables** | Pages 1-23 |
| --- | --- |
| **Supplemental figures captions** | Pages 24 |

| ***Sample*** | ***Year sampled*** | ***Morph*** | ***Sex*** | ***Weight 15dph*** | ***Weight 90dph*** |
| --- | --- | --- | --- | --- | --- |
| 1A | 2020 | grey | female | 285 | 490 |
| 2A | 2020 | brown | male | 330 | 455 |
| 3A | 2020 | grey | male | 290 | 460 |
| 4A | 2020 | grey | female | 325 | 535 |
| 6A | 2020 | brown | male | 290 | 455 |
| 7A | 2020 | grey | male | 320 | 435 |
| 8A | 2020 | grey | male | 320 | 435 |
| 9A | 2020 | brown | male | 365 | 465 |
| 11A | 2020 | grey | male | 300 | 455 |
| 12A | 2020 | brown | male | 240 | 450 |
| 13A | 2020 | brown | female | 265 | 525 |
| 14A | 2020 | grey | female | 410 | 475 |
| 15A | 2020 | grey | male | 335 | 400 |
| 16A | 2020 | brown | female | 370 | 525 |
| 17A | 2020 | grey | male | 320 | 435 |
| 18A | 2020 | brown | male | 320 | 405 |
| 18BA | 2021 | brown | male | 355 | 420 |
| 19A | 2021 | brown | male | 320 | 385 |
| 20A | 2020 | brown | male | 360 | 465 |
| 20BA | 2021 | brown | male | 330 | 415 |
| 26A | 2021 | brown | male | 305 | 440 |
| 28A | 2021 | grey | female | 430 | 465 |
| 29A | 2021 | grey | female | 400 | 505 |
| 30A | 2021 | brown | male | 320 | 395 |
| 31A | 2021 | grey | female | 355 | 520 |
| 32A | 2021 | grey | male | 315 | 430 |
| 33A | 2021 | brown | female | 370 | 510 |
| 34A | 2021 | grey | female | 415 | 470 |
| 35A | 2021 | brown | female | 290 | 490 |
| 36A | 2021 | grey | female | 280 | 465 |
| 37A | 2021 | brown | female | 350 | 470 |
| 41A | 2021 | grey | female | 300 | 470 |

Table S1 – Information on individual specimens utilized in this work.

All juveniles were collected at 15 post hatching from the wild and transported to the aviary. Information on this table relates to sex and colouration of the individuals utilized in this study. Individuals were numbered consecutively by year, thus overlap in years for the same number are denote as AB, as they represent different individuals. Weight (in grams) was measured at 15 and 90 days post hatching.

Table S2 – Statistics of transcripts mapping to reference with HISAT2

| ***Individual*** | ***reads*** | ***map%*** |
| --- | --- | --- |
| 11A | 46970815 | 80,11 |
| 12A | 46323665 | 66,71 |
| 13A | 46485554 | 70,75 |
| 14A | 46216181 | 70,19 |
| 15A | 43914314 | 75,65 |
| 16A | 41070531 | 71,14 |
| 17A | 46542697 | 73,31 |
| 18A | 46042512 | 71,51 |
| 18BA | 46781265 | 71,42 |
| 19A | 46862191 | 72,73 |
| 1A | 46300710 | 71,74 |
| 20A | 46709477 | 71,05 |
| 20BA | 46542014 | 71,93 |
| 4A | 47186388 | 82,72 |
| 6A | 46819170 | 81,07 |
| 26A | 46599117 | 70,74 |
| 27A | 47075148 | 74,45 |
| 28A | 46835747 | 70,23 |
| 29A | 46689694 | 70,58 |
| 2A | 46359038 | 69,95 |
| 30A | 38918542 | 70,35 |
| 31A | 46588852 | 69,25 |
| 32A | 46590388 | 71,68 |
| 33A | 46562490 | 73,27 |
| 34A | 45562249 | 71,49 |
| 35A | 46702014 | 71,81 |
| 36A | 46840946 | 70,48 |
| 37A | 41793440 | 71,02 |
| 41A | 46941297 | 77,43 |
| 8A | 46924001 | 80,17 |
| 7A | 46770072 | 70,7 |
| 9A | 46604356 | 69,59 |

Table S3 – Differentially expressed genes identified in this study in relation to location of orthologues in the chicken genome version (GRCg7b)

| **Gene Name** | **Ensembl ID** | **Chromosome** | **sex** |
| --- | --- | --- | --- |
| RELN | ENSGALG00010009720 | 1 | female |
| PTPRZ1 | ENSGALG00010001248 | 1 | male |
| WIF1 | ENSGALG00010012361 | 1 | male |
| SNAPC3 | ENSGALG00010013558 | 1 | male |
| ANKRD10 | ENSGALG00010013110 | 1 | male |
| TYR | ENSGALG00010007864 | 1 | male |
| RAB38 | ENSGALG00010007506 | 1 | male |
| TFAP2A | ENSGALG00010009668 | 2 | male |
| CDH6 | ENSGALG00010011040 | 2 | male |
| CNGB3 | ENSGALG00010010863 | 2 | female |
| EFEMP1 | ENSGALG00010018885 | 3 | male |
| BCL11A | ENSGALG00010017162 | 3 | male |
| ATP6V1C2 | ENSGALG00010002590 | 3 | male |
| ANKRD66 | ENSGALG00010008299 | 3 | male |
| FGF16 | ENSGALG00010013552 | 4 | male |
| EGF | ENSGALG00010003806 | 4 | female |
| ZGRF1 | ENSGALG00015016131 | 4 | female |
| MTNR1A | ENSGALG00010013336 | 4 | female |
| NKX2-1 | ENSGALG00010004654 | 5 | female |
| CEP128 | ENSGALG00010004346 | 5 | female |
| RAB11FIP2 | ENSGALG00010001958 | 6 | female |
| CTLA4 | ENSGALG00010021983 | 7 | female |
| RPAP2 | ENSGALG00010021932 | 8 | female |
| GPBP1L1 | ENSGALG00010026321 | 8 | male |
| USP13 | ENSGALG00010018123 | 9 | female |
| WWOX | ENSGALG00010007789 | 11 | female |
| FGD5 | ENSGALG00010022028 | 12 | female |
| FBF1 | ENSGALG00010030109 | 18 | male |
| ACSF2 | ENSGALG00010029650 | 18 | male |
| FAM64A | ENSGALG00010029292 | 19 | female |
| CEP250 | ENSGALG00010021373 | 20 | male |
| WFDC2 | ENSGALG00010015822 | 20 | male |
| TNFRSF18 | ENSGALG00010021538 | 21 | female |
| no_name | ENSGALG00010028742 | 25 | male |
| SELENBP1 | ENSGALG00010027444 | 25 | male |
| EEF2 | ENSGALG00010027732 | 28 | male |
| ACAA2 | ENSGALG00010010463 | Z | male |
| RPL17 | ENSGALG00010011192 | Z | male |
| DYM | ENSGALG00010010922 | Z | male |
| CTIF | ENSGALG00010010007 | Z | male |
| SMAD2 | ENSGALG00010010623 | Z | male |
| IER3IP1 | ENSGALG00010011367 | Z | male |
| PIAS2 | ENSGALG00010009774 | Z | male |
| LOXHD1 | ENSGALG00010012046 | Z | male |
| RNF165 | ENSGALG00010012051 | Z | male |
| HAUS1 | ENSGALG00010012082 | Z | male |
| ATP5F1AZ | ENSGALG00010012088 | Z | male |
| EPG5 | ENSGALG00010012119 | Z | male |
| SETBP1 | ENSGALG00010012148 | Z | male |
| PIK3C3 | ENSGALG00010009548 | Z | male |
| KIAA1328 | ENSGALG00010011456 | Z | male |
| TPGS2 | ENSGALG00010011458 | Z | male |
| UBAP2 | ENSGALG00010011429 | Z | male |
| IFNW1 | ENSGALG00010012349 | Z | male |
| UBAP1 | ENSGALG00010011846 | Z | male |
| FAM219A | ENSGALG00010010645 | Z | male |
| DCTN3 | ENSGALG00010009167 | Z | male |
| VCP | ENSGALG00010011335 | Z | male |
| RUSC2 | ENSGALG00010009246 | Z | male |
| HINT1 | ENSGALG00010009594 | Z | male |
| TPM2 | ENSGALG00010010970 | Z | male |
| CA9 | ENSGALG00010012303 | Z | male |
| PDZD2 | ENSGALG00010015647 | Z | male |
| GOLPH3 | ENSGALG00010014877 | Z | male |
| no_name | ENSGALG00010014907 | Z | male |
| SUB1 | ENSGALG00010012449 | Z | male |
| TARS | ENSGALG00010013116 | Z | male |
| AMACR | ENSGALG00010013203 | Z | male |
| RAD1 | ENSGALG00010013238 | Z | male |
| DNAJC21 | ENSGALG00010013264 | Z | male |
| IL7R | ENSGALG00010012726 | Z | male |
| LMBRD2 | ENSGALG00010013514 | Z | male |
| NADK2 | ENSGALG00010015616 | Z | male |
| WDR70 | ENSGALG00010012861 | Z | male |
| OSMR | ENSGALG00010013344 | Z | male |
| RICTOR | ENSGALG00010013357 | Z | male |
| TTC33 | ENSGALG00010014371 | Z | male |
| PRKAA1 | ENSGALG00010014397 | Z | male |
| RPL37 | ENSGALG00010013438 | Z | male |
| OXCT1 | ENSGALG00010012511 | Z | male |
| FBXO4 | ENSGALG00010012536 | Z | male |
| GHR | ENSGALG00010014614 | Z | male |
| HMGCS1 | ENSGALG00010014334 | Z | male |
| C5orf34 | ENSGALG00010014476 | Z | male |
| PAIP1 | ENSGALG00010014539 | Z | male |
| NNT | ENSGALG00010014579 | Z | male |
| PARP8 | ENSGALG00010014014 | Z | male |
| MOCS2 | ENSGALG00010012796 | Z | male |
| NDUFS4 | ENSGALG00010012578 | Z | male |
| ARL15 | ENSGALG00010014992 | Z | male |
| SNX18 | ENSGALG00010015013 | Z | male |
| CDC20B | ENSGALG00010015114 | Z | male |
| DHX29 | ENSGALG00010013953 | Z | male |
| SKIV2L2 | ENSGALG00010014041 | Z | male |
| SLC38A9 | ENSGALG00010014167 | Z | male |
| MAP3K1 | ENSGALG00010013033 | Z | male |
| PLK2 | ENSGALG00010015163 | Z | male |
| PDE4D | ENSGALG00010015196 | Z | male |
| DEPDC1B | ENSGALG00010013468 | Z | male |
| ERCC8 | ENSGALG00010012669 | Z | male |
| NDUFAF2 | ENSGALG00010012688 | Z | male |
| ZSWIM6 | ENSGALG00010012722 | Z | male |
| KIF2A | ENSGALG00010014736 | Z | male |
| DIMT1 | ENSGALG00010014754 | Z | male |
| CWC27 | ENSGALG00010012154 | Z | male |
| CENPK | ENSGALG00010012196 | Z | male |
| PPWD1 | ENSGALG00010012210 | Z | male |
| TRIM23 | ENSGALG00010012247 | Z | male |
| TRAPPC13 | ENSGALG00010012309 | Z | male |
| SGTB | ENSGALG00010012326 | Z | male |
| NLN | ENSGALG00010012346 | Z | male |
| MAST4 | ENSGALG00010010381 | Z | male |
| PIK3R1 | ENSGALG00010011922 | Z | male |
| SLC30A5 | ENSGALG00010010751 | Z | male |
| MRPS36 | ENSGALG00010010834 | Z | male |
| CDK7 | ENSGALG00010010937 | Z | male |
| SERINC5 | ENSGALG00010011515 | Z | male |
| CMYA5 | ENSGALG00010012414 | Z | male |
| HOMER1 | ENSGALG00010012521 | Z | male |
| JMY | ENSGALG00010012535 | Z | male |
| ARSB | ENSGALG00010013306 | Z | male |
| LHFPL2 | ENSGALG00010012614 | Z | male |
| SCAMP1 | ENSGALG00010012667 | Z | male |
| AP3B1 | ENSGALG00010012751 | Z | male |
| TBCA | ENSGALG00010012795 | Z | male |
| WDR41 | ENSGALG00010010254 | Z | male |
| PDE8B | ENSGALG00010010275 | Z | male |
| AGGF1 | ENSGALG00010010457 | Z | male |
| S100Z | ENSGALG00010011454 | Z | male |
| IQGAP2 | ENSGALG00010010688 | Z | male |
| ANKDD1B | ENSGALG00010010962 | Z | male |
| POLK | ENSGALG00010010980 | Z | male |
| CERT1 | ENSGALG00010010985 | Z | male |
| HMGCR | ENSGALG00010011029 | Z | male |
| NSA2 | ENSGALG00010010713 | Z | male |
| GFM2 | ENSGALG00010010733 | Z | male |
| HEXB | ENSGALG00010010769 | Z | male |
| ENC1 | ENSGALG00010010905 | Z | male |
| ARHGEF28 | ENSGALG00010011724 | Z | male |
| UTP15 | ENSGALG00010011747 | Z | male |
| ANKRA2 | ENSGALG00010011774 | Z | male |
| BTF3 | ENSGALG00010010447 | Z | male |
| TNPO1 | ENSGALG00010010186 | Z | male |
| PTCD2 | ENSGALG00010011572 | Z | male |
| MRPS27 | ENSGALG00010011649 | Z | male |
| CBWD1 | ENSGALG00010011287 | Z | male |
| SMARCA2 | ENSGALG00010011808 | Z | male |
| VLDLR | ENSGALG00010011837 | Z | female |
| PUM3 | ENSGALG00010011384 | Z | male |
| RFX3 | ENSGALG00010011873 | Z | male |
| RCL1 | ENSGALG00010011222 | Z | male |
| RIC1 | ENSGALG00010012889 | Z | male |
| MLANA | ENSGALG00010012923 | Z | male |
| UHRF2 | ENSGALG00010012965 | Z | male |
| KDM4C | ENSGALG00010011253 | Z | male |
| PTPRD | ENSGALG00010012063 | Z | male |
| TYRP1 | ENSGALG00010014092 | Z | male |
| LURAP1L | ENSGALG00010016139 | Z | male |
| NFIB | ENSGALG00010016418 | Z | male |
| ZDHHC21 | ENSGALG00010016445 | Z | male |
| TTC39B | ENSGALG00010016468 | Z | male |
| PSIP1 | ENSGALG00010014035 | Z | male |
| CCDC171 | ENSGALG00010014372 | Z | female |
| BNC2 | ENSGALG00010016535 | Z | male |
| CNTLN | ENSGALG00010016566 | Z | male |
| ADAMTSL1 | ENSGALG00010016043 | Z | male |
| HAUS6 | ENSGALG00010016114 | Z | male |
| DENND4C | ENSGALG00010016305 | Z | male |
| RPS6 | ENSGALG00010016308 | Z | male |
| FOCAD | ENSGALG00010014153 | Z | male |
| TJP2 | ENSGALG00010016887 | Z | male |
| APBA1 | ENSGALG00010014837 | Z | male |
| PTAR1 | ENSGALG00010014914 | Z | male |
| SMC5 | ENSGALG00010014971 | Z | male |
| KLF9 | ENSGALG00010014987 | Z | male |
| C9orf85 | ENSGALG00010014220 | Z | male |
| ANXA1 | ENSGALG00010014114 | Z | male |
| OSTF1 | ENSGALG00010015493 | Z | male |
| RFK | ENSGALG00010015064 | Z | male |
| VPS13A | ENSGALG00010015181 | Z | male |
| GNAQ | ENSGALG00010015212 | Z | male |
| CEP78 | ENSGALG00010015894 | Z | male |
| PSAT1 | ENSGALG00010015533 | Z | male |
| TLE4 | ENSGALG00010015946 | Z | male |
| TLE1 | ENSGALG00010015766 | Z | male |
| RASEF | ENSGALG00010015541 | Z | male |
| IDNK | ENSGALG00010015621 | Z | male |
| UBQLN1 | ENSGALG00010015628 | Z | male |
| HNRNPK | ENSGALG00010014677 | Z | male |
| NTRK2 | ENSGALG00010010150 | Z | male |
| AGTPBP1 | ENSGALG00010008636 | Z | male |
| NAA35 | ENSGALG00010008699 | Z | male |
| GOLM1 | ENSGALG00010008752 | Z | male |
| ISCA1 | ENSGALG00010008213 | Z | male |
| ZCCHC6 | ENSGALG00010008231 | Z | male |
| DAPK1 | ENSGALG00010009744 | Z | male |
| CTSV | ENSGALG00010010311 | Z | male |
| PTCH1 | ENSGALG00010010406 | Z | male |
| ERCC6L2 | ENSGALG00010010064 | Z | male |
| HSD17B3 | ENSGALG00010008777 | Z | male |
| CDC14B | ENSGALG00010010844 | Z | male |
| SPIN1 | ENSGALG00010009031 | Z | male |
| SECISBP2 | ENSGALG00010009153 | Z | male |
| SEMA4D | ENSGALG00010009199 | Z | male |
| AUH | ENSGALG00010009396 | Z | male |
| SPTLC1 | ENSGALG00010009464 | Z | male |
| CDC42SE2 | ENSGALG00010009492 | Z | male |
| CHSY3 | ENSGALG00010009606 | Z | male |
| ADAMTS19 | ENSGALG00010009683 | Z | male |
| YTHDC2 | ENSGALG00010008243 | Z | male |
| MCC | ENSGALG00010008259 | Z | male |
| DCP2 | ENSGALG00010008272 | Z | male |
| REEP5 | ENSGALG00010008292 | Z | male |
| APC | ENSGALG00010008333 | Z | male |
| EPB41L4A | ENSGALG00010010011 | Z | male |
| WDR36 | ENSGALG00010010302 | Z | male |
| SLC25A46 | ENSGALG00010009617 | Z | male |
| MAN2A1 | ENSGALG00010009762 | Z | male |
| FER | ENSGALG00010009663 | Z | male |
| EFNA5 | ENSGALG00010010266 | Z | male |
| PPIP5K2 | ENSGALG00010008424 | Z | male |
| GIN1 | ENSGALG00010008450 | Z | male |
| CHD1 | ENSGALG00010012470 | Z | male |
| RGMB | ENSGALG00010012903 | Z | male |
| RIOK2 | ENSGALG00010011881 | Z | male |
| LNPEP | ENSGALG00010013246 | Z | male |
| SHB | ENSGALG00010013253 | Z | male |
| RNF38 | ENSGALG00010012095 | Z | male |
| NANS | ENSGALG00010012250 | Z | male |
| CLTA | ENSGALG00010013183 | Z | male |
| GNE | ENSGALG00010013225 | Z | male |
| MFSD7 | ENSGALG00010012974 | Z | male |
| PIGG | ENSGALG00010012353 | Z | male |
| GAK | ENSGALG00010013313 | Z | male |
| TMEM175 | ENSGALG00010013431 | Z | male |
| ATP5I | ENSGALG00010013446 | Z | male |
| IDUA | ENSGALG00010013546 | Z | male |
| RNF170 | ENSGALG00010011971 | Z | male |
| HOOK3 | ENSGALG00010012011 | Z | male |
| PSD3 | ENSGALG00010014032 | Z | male |
| SLC44A1 | ENSGALG00010014184 | Z | male |
| FKTN | ENSGALG00010013065 | Z | male |
| ZNF462 | ENSGALG00010014265 | Z | male |
| RAD23B | ENSGALG00010014317 | Z | male |
| KLF4 | ENSGALG00010014323 | Z | male |
| PHAX | ENSGALG00010012574 | Z | male |
| no_name | ENSGALG00010012893 | Z | male |
| CAST | ENSGALG00010012190 | Z | male |
| ARSK | ENSGALG00010013117 | Z | male |
| TTC37 | ENSGALG00010013132 | Z | male |
| FAM172A | ENSGALG00010013497 | Z | male |
| ARRDC3 | ENSGALG00010012830 | Z | male |
| CETN3 | ENSGALG00010013696 | Z | male |
| TMEM161B | ENSGALG00010005477 | Z | male |
| CCNH | ENSGALG00010005887 | Z | male |
| COX7C | ENSGALG00010006252 | Z | male |
| XRCC4 | ENSGALG00010006821 | Z | male |
| TMEM167A | ENSGALG00010006851 | Z | male |
| RPS23 | ENSGALG00010006178 | Z | male |
| ATG10 | ENSGALG00010006223 | Z | male |
| SSBP2 | ENSGALG00010006575 | Z | male |
| MSH3 | ENSGALG00010007217 | Z | male |
| FAM151B | ENSGALG00010007277 | Z | male |
| no_name | ENSGALG00010007284 | Z | male |
| RNF20 | ENSGALG00010005549 | Z | male |
| MRPL50 | ENSGALG00010005594 | Z | male |
| PALM2AKAP2 | ENSGALG00010006957 | Z | male |
| INIP | ENSGALG00010007066 | Z | male |
| HSDL2 | ENSGALG00010006029 | Z | male |
| UGCG | ENSGALG00010005963 | Z | male |
| DNAJC25 | ENSGALG00010006283 | Z | male |
| ECPAS | ENSGALG00010006287 | Z | male |
| SMC2 | ENSGALG00010006345 | Z | male |
| PTGR1 | ENSGALG00010006355 | Z | male |
| TXN | ENSGALG00010005495 | Z | male |
| SVEP1 | ENSGALG00010005772 | Z | male |
| LPAR1 | ENSGALG00010005828 | Z | male |
| TOPORS | ENSGALG00010005637 | Z | male |
| CAAP1 | ENSGALG00010006378 | Z | male |
| PLAA | ENSGALG00010006465 | Z | male |
| IFT74 | ENSGALG00010006480 | Z | male |
| MOB3B | ENSGALG00010006544 | Z | male |
| C9orf72 | ENSGALG00010006249 | Z | male |
| ELP1 | ENSGALG00010008136 | Z | male |
| APTX | ENSGALG00010008141 | Z | male |
| DNAJA1 | ENSGALG00010008149 | Z | male |
| SMU1 | ENSGALG00010008153 | Z | male |
| B4GALT1 | ENSGALG00010009486 | Z | male |
| XPA | ENSGALG00010009534 | Z | male |
| NCBP1 | ENSGALG00010009593 | Z | male |
| TDRD7 | ENSGALG00010009635 | Z | male |
| HSD17B4 | ENSGALG00010009280 | Z | male |
| TNFAIP8 | ENSGALG00010009360 | Z | male |
| DMXL1 | ENSGALG00010009367 | Z | male |
| DTWD2 | ENSGALG00010009236 | Z | male |
| SEMA6A | ENSGALG00010008179 | Z | male |
| AP3S1 | ENSGALG00010009103 | Z | male |
| ATG12 | ENSGALG00010009120 | Z | male |
| MCCC2 | ENSGALG00010009143 | Z | male |
| MTAP | ENSGALG00010001141 | Z | male |
| PGGT1B | ENSGALG00010001147 | Z | male |
| FEM1C | ENSGALG00010001152 | Z | male |
| ALDH7A1 | ENSGALG00010001156 | Z | male |
| SRFBP1 | ENSGALG00010001286 | Z | male |
| SNX2 | ENSGALG00010001204 | Z | male |
| PPIC | ENSGALG00010001213 | Z | male |
| CEP120 | ENSGALG00010001306 | Z | male |
| POLR1E | ENSGALG00010001171 | Z | male |
| ZCCHC7 | ENSGALG00010001182 | Z | male |

Table S4 – All differentially expressed genes and respective statistics. Genes were retained for a p-value (adj) < 0.1.

| **gene_name** | **baseMean** | **log2FoldChange** | **lfcSE** | **stat** | **pvalue** | **padj** |
| --- | --- | --- | --- | --- | --- | --- |
| ZGRF1 | 88.26532 | 7.848608 | 0.646737 | 12.1357 | 6.83E-34 | 9.23E-30 |
| **VLDLR** | **32.81673** | **7.105816** | **0.723635** | **9.819619** | **9.27E-23** | **4.17E-19** |
| DUT1 | 7.590533 | 4.972376 | 1.321373 | 3.763038 | 0.000168 | 0.009859 |
| ERVMER34-1 | 21.84995 | 4.883137 | 0.787018 | 6.204606 | 5.48E-10 | 1.95E-07 |
| USP13 | 5.786679 | 3.629023 | 0.980962 | 3.699453 | 0.000216 | 0.012263 |
| **CCDC171** | **12.94893** | **3.485482** | **0.811046** | **4.297514** | **1.73E-05** | **0.001467** |
| EGF | 9.252528 | 3.208629 | 0.785878 | 4.082857 | 4.45E-05 | 0.003213 |
| TONSL | 4.235951 | 2.756271 | 0.893535 | 3.08468 | 0.002038 | 0.08495 |
| **CDH6** | **195.6747** | **2.640113** | **0.766385** | **3.444893** | **0.000571** | **0.028482** |
| CNGB3 | 90.24741 | 2.430127 | 0.602546 | 4.033096 | 5.5E-05 | 0.003893 |
| TNFRSF18 | 25.28397 | 2.368522 | 0.76661 | 3.089606 | 0.002004 | 0.084078 |
| MTNR1A | 35.4836 | 2.300181 | 0.633805 | 3.629162 | 0.000284 | 0.015545 |
| FAM64A | 63.53967 | 2.29713 | 0.573782 | 4.003489 | 6.24E-05 | 0.004346 |
| GPR128 | 250.9939 | 1.161931 | 0.350202 | 3.317888 | 0.000907 | 0.042839 |
| CTLA4 | 382.4904 | 1.092451 | 0.33995 | 3.213569 | 0.001311 | 0.058252 |
| RELN | 296.7732 | 0.977846 | 0.293958 | 3.326477 | 0.00088 | 0.041686 |
| RAB11FIP2 | 1690.712 | 0.793027 | 0.221842 | 3.574739 | 0.000351 | 0.018571 |
| CEP128 | 1960.616 | 0.639564 | 0.174313 | 3.669043 | 0.000243 | 0.01345 |
| FGD5 | 1616.673 | 0.636672 | 0.166689 | 3.819533 | 0.000134 | 0.008099 |
| RPAP2 | 1128.417 | 0.533277 | 0.175706 | 3.035052 | 0.002405 | 0.095339 |
| SDCCAG8 | 2119.949 | 0.432477 | 0.142769 | 3.029198 | 0.002452 | 0.096285 |
| WWOX | 6218.475 | 0.412032 | 0.116295 | 3.542985 | 0.000396 | 0.020541 |
| **ANKRD10** | **8529.263** | **0.381884** | **0.126008** | **3.030631** | **0.00244** | **0.096167** |
| **FBF1** | **1917.092** | **-0.45629** | **0.139364** | **-3.27408** | **0.00106** | **0.049208** |
| NIPBL | 5657.249 | -0.48759 | 0.115367 | -4.22638 | 2.37E-05 | 0.001876 |
| VPS13A | 6915.768 | -0.58519 | 0.165327 | -3.5396 | 0.000401 | 0.020661 |
| TFAP2A | 1891.912 | -0.61352 | 0.199016 | -3.08279 | 0.002051 | 0.084973 |
| ZFYVE16 | 1699.118 | -0.63209 | 0.160292 | -3.94336 | 8.03E-05 | 0.005269 |
| SPIN1 | 1356.181 | -0.64219 | 0.161185 | -3.98416 | 6.77E-05 | 0.004597 |
| HSDL2 | 914.6058 | -0.64292 | 0.177361 | -3.62493 | 0.000289 | 0.015618 |
| TNPO1 | 2375.941 | -0.64431 | 0.163134 | -3.9496 | 7.83E-05 | 0.005158 |
| BNC2 | 16369.68 | -0.64602 | 0.203645 | -3.17229 | 0.001512 | 0.065576 |
| SUB1 | 614.5238 | -0.66094 | 0.177834 | -3.71662 | 0.000202 | 0.011557 |
| ERBB2IP | 5358.062 | -0.66142 | 0.125158 | -5.2847 | 1.26E-07 | 2.16E-05 |
| AP3S1 | 1388.183 | -0.66557 | 0.196333 | -3.38998 | 0.000699 | 0.033842 |
| TJP2 | 5977.886 | -0.67232 | 0.21281 | -3.15923 | 0.001582 | 0.067835 |
| KIAA0368 | 2549.859 | -0.68473 | 0.115943 | -5.90573 | 3.51E-09 | 1.01E-06 |
| ZCCHC7 | 1326.518 | -0.68643 | 0.218707 | -3.13859 | 0.001698 | 0.072112 |
| B4GALT1 | 2106.154 | -0.6892 | 0.177676 | -3.87896 | 0.000105 | 0.006591 |
| PPIC | 759.9021 | -0.69079 | 0.228621 | -3.02154 | 0.002515 | 0.097619 |
| ZFR | 1562.988 | -0.70043 | 0.134183 | -5.21996 | 1.79E-07 | 2.88E-05 |
| TTC33 | 1116.165 | -0.70906 | 0.215236 | -3.29431 | 0.000987 | 0.046115 |
| APC | 4285.483 | -0.71154 | 0.164126 | -4.33532 | 1.46E-05 | 0.001285 |
| SMAD2 | 1206.461 | -0.71331 | 0.203874 | -3.49879 | 0.000467 | 0.023824 |
| MAN2A1 | 1659.481 | -0.71576 | 0.137783 | -5.19487 | 2.05E-07 | 3.18E-05 |
| ZSWIM6 | 5037.768 | -0.71843 | 0.180235 | -3.98607 | 6.72E-05 | 0.004583 |
| SLC38A9 | 1603.153 | -0.71969 | 0.18078 | -3.98104 | 6.86E-05 | 0.004611 |
| ACAA2 | 1367.372 | -0.72031 | 0.199402 | -3.61233 | 0.000303 | 0.016266 |
| MAP3K1 | 14337.07 | -0.72905 | 0.182721 | -3.98999 | 6.61E-05 | 0.004531 |
| RICTOR | 3477.31 | -0.72917 | 0.130239 | -5.59874 | 2.16E-08 | 4.86E-06 |
| PLK2 | 2332.906 | -0.7292 | 0.227697 | -3.20252 | 0.001362 | 0.060335 |
| PRKAA1 | 1471.835 | -0.73957 | 0.149468 | -4.948 | 7.5E-07 | 0.0001 |
| **FER** | **4175.909** | **-0.74095** | **0.132553** | **-5.58984** | **2.27E-08** | **4.87E-06** |
| ATG10 | 1228.51 | -0.75631 | 0.200795 | -3.76655 | 0.000166 | 0.009763 |
| FCHO2 | 3020.912 | -0.75648 | 0.140879 | -5.36971 | 7.89E-08 | 1.48E-05 |
| PIK3R1 | 2739.602 | -0.75932 | 0.171351 | -4.43137 | 9.36E-06 | 0.000897 |
| SVEP1 | 1452.17 | -0.76006 | 0.209547 | -3.62717 | 0.000287 | 0.015545 |
| SNX18 | 668.8729 | -0.76081 | 0.206087 | -3.69171 | 0.000223 | 0.01259 |
| FBXO4 | 281.3274 | -0.76223 | 0.206721 | -3.68725 | 0.000227 | 0.012759 |
| RASEF | 706.9139 | -0.76286 | 0.242453 | -3.14641 | 0.001653 | 0.070432 |
| DENND4C | 4366.441 | -0.76582 | 0.124673 | -6.14267 | 8.11E-10 | 2.67E-07 |
| KIF2A | 2150.672 | -0.78294 | 0.200375 | -3.90737 | 9.33E-05 | 0.005919 |
| CAST | 6479.392 | -0.78576 | 0.175707 | -4.47197 | 7.75E-06 | 0.000759 |
| IFT74 | 751.9906 | -0.79012 | 0.171237 | -4.6142 | 3.95E-06 | 0.000423 |
| BDP1 | 4169.428 | -0.79513 | 0.173038 | -4.59511 | 4.33E-06 | 0.000453 |
| HMGCR | 2770.997 | -0.79547 | 0.136859 | -5.81233 | 6.16E-09 | 1.63E-06 |
| SNX2 | 2601.692 | -0.79593 | 0.117514 | -6.7731 | 1.26E-11 | 8.51E-09 |
| HNRNPK | 3011.789 | -0.79615 | 0.159893 | -4.97926 | 6.38E-07 | 8.8E-05 |
| UBQLN1 | 1609.956 | -0.79623 | 0.152211 | -5.23112 | 1.68E-07 | 2.74E-05 |
| TDRD7 | 956.5345 | -0.79626 | 0.260702 | -3.05429 | 0.002256 | 0.091786 |
| GAK | 2480.256 | -0.79641 | 0.188033 | -4.23546 | 2.28E-05 | 0.001812 |
| TTC39B | 6188.547 | -0.79938 | 0.214526 | -3.72627 | 0.000194 | 0.011266 |
| AUH | 3325.471 | -0.80333 | 0.260919 | -3.07886 | 0.002078 | 0.085838 |
| CWC27 | 3182.692 | -0.80544 | 0.16172 | -4.98046 | 6.34E-07 | 8.8E-05 |
| PPWD1 | 381.1343 | -0.80964 | 0.256178 | -3.16046 | 0.001575 | 0.067763 |
| RPS23 | 15564.14 | -0.81288 | 0.179909 | -4.5183 | 6.23E-06 | 0.000619 |
| NCBP1 | 1144.54 | -0.81358 | 0.205345 | -3.96204 | 7.43E-05 | 0.004921 |
| SKIV2L2 | 1741.616 | -0.81367 | 0.153432 | -5.30313 | 1.14E-07 | 2.02E-05 |
| BC067074 | 1159.481 | -0.81483 | 0.248561 | -3.27818 | 0.001045 | 0.048666 |
| CHD1 | 2539.609 | -0.8194 | 0.185673 | -4.41312 | 1.02E-05 | 0.00096 |
| PPIP5K2 | 1093.849 | -0.81951 | 0.216602 | -3.78351 | 0.000155 | 0.009202 |
| NANS | 430.2534 | -0.82006 | 0.216307 | -3.79118 | 0.00015 | 0.008961 |
| TPM2 | 563.3675 | -0.82228 | 0.260151 | -3.16077 | 0.001574 | 0.067763 |
| PIAS2 | 1046.744 | -0.82336 | 0.1463 | -5.62787 | 1.82E-08 | 4.32E-06 |
| **TFAP2A** | **260.6164** | **-0.82484** | **0.266353** | **-3.09679** | **0.001956** | **0.082323** |
| DMXL1 | 8425.642 | -0.82658 | 0.160314 | -5.156 | 2.52E-07 | 3.79E-05 |
| S100Z | 1524.184 | -0.82698 | 0.24634 | -3.35707 | 0.000788 | 0.037733 |
| C9orf41 | 690.7203 | -0.82825 | 0.272036 | -3.04463 | 0.00233 | 0.093939 |
| FBXL17 | 9916.165 | -0.82874 | 0.126226 | -6.56549 | 5.19E-11 | 2.69E-08 |
| AKAP2 | 4340.693 | -0.83094 | 0.244738 | -3.39522 | 0.000686 | 0.033561 |
| CTSL | 1512.9 | -0.83144 | 0.155258 | -5.35519 | 8.55E-08 | 1.54E-05 |
| XRCC4 | 1570.497 | -0.83274 | 0.273396 | -3.04592 | 0.00232 | 0.093815 |
| CCNH | 685.0361 | -0.83913 | 0.195981 | -4.2817 | 1.85E-05 | 0.001555 |
| UBAP1 | 1406.987 | -0.84411 | 0.22234 | -3.79648 | 0.000147 | 0.008811 |
| KDM4C | 2630.719 | -0.84504 | 0.266215 | -3.17427 | 0.001502 | 0.065454 |
| PTAR1 | 1268.465 | -0.85163 | 0.144824 | -5.88046 | 4.09E-09 | 1.13E-06 |
| PTPRD | 821.8566 | -0.85591 | 0.278714 | -3.07091 | 0.002134 | 0.087887 |
| UBAP2 | 5024.388 | -0.85916 | 0.132937 | -6.46291 | 1.03E-10 | 4.78E-08 |
| PAPD4 | 1588.259 | -0.85925 | 0.200911 | -4.27678 | 1.9E-05 | 0.001557 |
| AGGF1 | 1153.855 | -0.85999 | 0.200911 | -4.28045 | 1.87E-05 | 0.001555 |
| PGGT1B | 1191.671 | -0.86272 | 0.15846 | -5.44439 | 5.2E-08 | 1.03E-05 |
| DHX29 | 2438.481 | -0.86362 | 0.160904 | -5.36733 | 7.99E-08 | 1.48E-05 |
| PDE4D | 3935.373 | -0.86448 | 0.28597 | -3.02298 | 0.002503 | 0.097539 |
| CDC20B | 328.1832 | -0.86476 | 0.267661 | -3.2308 | 0.001234 | 0.055397 |
| ARHGEF28 | 898.6216 | -0.86867 | 0.199824 | -4.34719 | 1.38E-05 | 0.001242 |
| ARL15 | 6150.945 | -0.86938 | 0.200197 | -4.3426 | 1.41E-05 | 0.00126 |
| UHRF2 | 1670.472 | -0.87006 | 0.164498 | -5.28915 | 1.23E-07 | 2.16E-05 |
| RIC1 | 3673.63 | -0.87066 | 0.187814 | -4.63574 | 3.56E-06 | 0.000384 |
| C5orf42 | 2616.964 | -0.87127 | 0.181885 | -4.79021 | 1.67E-06 | 0.000201 |
| ZCCHC6 | 2479.676 | -0.87156 | 0.208428 | -4.18159 | 2.89E-05 | 0.002222 |
| MCCC2 | 1948.105 | -0.87413 | 0.216991 | -4.02843 | 5.62E-05 | 0.00395 |
| EFNA5 | 34995.42 | -0.87432 | 0.174115 | -5.02153 | 5.13E-07 | 7.37E-05 |
| SLC25A46 | 667.7244 | -0.87453 | 0.210659 | -4.15139 | 3.3E-05 | 0.002508 |
| TMEM175 | 363.3463 | -0.87644 | 0.256225 | -3.4206 | 0.000625 | 0.03103 |
| OSTF1 | 1011.011 | -0.87855 | 0.2081 | -4.22177 | 2.42E-05 | 0.001904 |
| ZCCHC6 | 3874.403 | -0.87872 | 0.200557 | -4.38138 | 1.18E-05 | 0.001091 |
| CSNK1G3 | 1038.23 | -0.87956 | 0.179931 | -4.88832 | 1.02E-06 | 0.000128 |
| WDR41 | 984.9669 | -0.88342 | 0.219705 | -4.02092 | 5.8E-05 | 0.004057 |
| C9orf3 | 7366.784 | -0.88517 | 0.133667 | -6.62221 | 3.54E-11 | 1.99E-08 |
| VCP | 1368.381 | -0.88757 | 0.182556 | -4.86189 | 1.16E-06 | 0.000144 |
| ATP5A1 | 2700.256 | -0.88859 | 0.186297 | -4.76973 | 1.84E-06 | 0.00022 |
| PHAX | 910.6056 | -0.89041 | 0.288595 | -3.08531 | 0.002033 | 0.08495 |
| PTPLAD2 | 970.2814 | -0.89107 | 0.293615 | -3.03482 | 0.002407 | 0.095339 |
| SRFBP1 | 2618.708 | -0.89252 | 0.158788 | -5.62083 | 1.9E-08 | 4.43E-06 |
| C5orf63 | 437.9909 | -0.89259 | 0.274943 | -3.24647 | 0.001168 | 0.052788 |
| GFM2 | 1147.417 | -0.89502 | 0.169392 | -5.2837 | 1.27E-07 | 2.16E-05 |
| SCAMP1 | 2034.522 | -0.89539 | 0.169873 | -5.27093 | 1.36E-07 | 2.29E-05 |
| WDR36 | 1575.936 | -0.89562 | 0.228758 | -3.91512 | 9.04E-05 | 0.00584 |
| MYO5B | 3097.423 | -0.89656 | 0.253661 | -3.53446 | 0.000409 | 0.020907 |
| REEP5 | 2549.131 | -0.89765 | 0.225397 | -3.98254 | 6.82E-05 | 0.004605 |
| ERCC6L2 | 1630.907 | -0.8982 | 0.295376 | -3.04087 | 0.002359 | 0.094835 |
| UTP15 | 976.025 | -0.89949 | 0.252812 | -3.55795 | 0.000374 | 0.019569 |
| LPAR1 | 2370.762 | -0.90046 | 0.181077 | -4.97282 | 6.6E-07 | 9E-05 |
| NADK2 | 1366.33 | -0.90353 | 0.227333 | -3.97447 | 7.05E-05 | 0.004708 |
| SHB | 715.0329 | -0.90466 | 0.269642 | -3.35505 | 0.000794 | 0.037875 |
| HOMER1 | 1213.822 | -0.90729 | 0.243475 | -3.72642 | 0.000194 | 0.011266 |
| CEP78 | 841.9849 | -0.90886 | 0.2238 | -4.06103 | 4.89E-05 | 0.00351 |
| IER3IP1 | 379.9549 | -0.91007 | 0.249336 | -3.64996 | 0.000262 | 0.014402 |
| HSD17B4 | 4047.449 | -0.9114 | 0.237861 | -3.83163 | 0.000127 | 0.007746 |
| MAST4 | 9016.109 | -0.91282 | 0.140683 | -6.48848 | 8.67E-11 | 4.34E-08 |
| RASA1 | 334.9424 | -0.91284 | 0.264987 | -3.44484 | 0.000571 | 0.028482 |
| AMACR | 712.7318 | -0.91301 | 0.217653 | -4.1948 | 2.73E-05 | 0.002108 |
| ARRDC3 | 6854.738 | -0.91566 | 0.194662 | -4.70384 | 2.55E-06 | 0.000289 |
| HAUS6 | 1273.743 | -0.91597 | 0.233788 | -3.91795 | 8.93E-05 | 0.0058 |
| OSMR | 1776.519 | -0.91788 | 0.223834 | -4.1007 | 4.12E-05 | 0.003027 |
| ARSB | 538.2951 | -0.91931 | 0.254198 | -3.6165 | 0.000299 | 0.01607 |
| IDNK | 785.3431 | -0.91967 | 0.305188 | -3.01344 | 0.002583 | 0.099124 |
| CEP120 | 870.7135 | -0.92214 | 0.213994 | -4.30917 | 1.64E-05 | 0.00141 |
| GOLPH3 | 1269.733 | -0.92346 | 0.131537 | -7.02053 | 2.21E-12 | 2.3E-09 |
| SSBP2 | 12586.32 | -0.92367 | 0.160595 | -5.75154 | 8.84E-09 | 2.17E-06 |
| HSD17B3 | 1738.085 | -0.92371 | 0.234959 | -3.93137 | 8.45E-05 | 0.005512 |
| SERINC5 | 2879.835 | -0.92636 | 0.176174 | -5.25819 | 1.45E-07 | 2.43E-05 |
| ENC1 | 362.3031 | -0.9282 | 0.306292 | -3.03045 | 0.002442 | 0.096167 |
| EPG5 | 1833.978 | -0.9301 | 0.233057 | -3.99086 | 6.58E-05 | 0.004531 |
| AP3B1 | 3949.316 | -0.93026 | 0.135644 | -6.8581 | 6.98E-12 | 5.89E-09 |
| PLAA | 1512.099 | -0.93345 | 0.129231 | -7.22307 | 5.08E-13 | 7.24E-10 |
| VCP | 758.9198 | -0.93415 | 0.203612 | -4.58788 | 4.48E-06 | 0.000462 |
| ZDHHC21 | 1184.096 | -0.93424 | 0.205136 | -4.55424 | 5.26E-06 | 0.000526 |
| GOLM1 | 1310.244 | -0.93602 | 0.230574 | -4.05951 | 4.92E-05 | 0.003515 |
| TMEM161B | 1249.821 | -0.93857 | 0.179167 | -5.23855 | 1.62E-07 | 2.67E-05 |
| HOOK3 | 14330.98 | -0.94291 | 0.152566 | -6.18035 | 6.4E-10 | 2.22E-07 |
| SERF1A | 422.0584 | -0.943 | 0.305792 | -3.08378 | 0.002044 | 0.08495 |
| AGTPBP1 | 2304.782 | -0.94367 | 0.205599 | -4.58986 | 4.44E-06 | 0.000461 |
| PDE8B | 1510.983 | -0.94382 | 0.141854 | -6.65349 | 2.86E-11 | 1.76E-08 |
| ANKRA2 | 454.3429 | -0.94399 | 0.245134 | -3.85091 | 0.000118 | 0.007226 |
| YTHDC2 | 1219.753 | -0.9447 | 0.172549 | -5.47493 | 4.38E-08 | 8.82E-06 |
| DYM | 3646.397 | -0.948 | 0.154101 | -6.15182 | 7.66E-10 | 2.59E-07 |
| PSIP1 | 2257.042 | -0.9517 | 0.122358 | -7.778 | 7.37E-15 | 2.49E-11 |
| GIN1 | 551.1024 | -0.95229 | 0.243632 | -3.90873 | 9.28E-05 | 0.005919 |
| DNAJC21 | 755.4767 | -0.9542 | 0.294356 | -3.24166 | 0.001188 | 0.053508 |
| IKBKAP | 487.8319 | -0.9581 | 0.317629 | -3.01641 | 0.002558 | 0.098436 |
| C9orf72 | 729.9722 | -0.96082 | 0.258382 | -3.71859 | 0.0002 | 0.011516 |
| MSH3 | 1356.37 | -0.96198 | 0.191188 | -5.03159 | 4.86E-07 | 7.07E-05 |
| RIOK2 | 1390.881 | -0.9663 | 0.187154 | -5.16314 | 2.43E-07 | 3.69E-05 |
| TMEM167A | 712.7781 | -0.96758 | 0.132471 | -7.30408 | 2.79E-13 | 5.39E-10 |
| SLC30A5 | 1133.017 | -0.97138 | 0.206651 | -4.70058 | 2.59E-06 | 0.00029 |
| **SNAPC3** | **994.9815** | **-0.97264** | **0.20414** | **-4.76457** | **1.89E-06** | **0.000222** |
| MRPS27 | 1435.853 | -0.97495 | 0.237224 | -4.10985 | 3.96E-05 | 0.002955 |
| TNFAIP8 | 922.1375 | -0.97771 | 0.263391 | -3.71201 | 0.000206 | 0.011719 |
| PAIP1 | 1928.455 | -0.97911 | 0.153899 | -6.36202 | 1.99E-10 | 8.41E-08 |
| SETBP1 | 6805.977 | -0.97937 | 0.177702 | -5.51131 | 3.56E-08 | 7.29E-06 |
| IQGAP2 | 3775.364 | -0.97978 | 0.21128 | -4.63733 | 3.53E-06 | 0.000384 |
| FAM172A | 3151.177 | -0.98042 | 0.131515 | -7.45487 | 9E-14 | 2.03E-10 |
| SMU1 | 721.4445 | -0.98058 | 0.208521 | -4.70256 | 2.57E-06 | 0.000289 |
| POLK | 1688.188 | -0.9814 | 0.189155 | -5.18834 | 2.12E-07 | 3.26E-05 |
| JMY | 3908.306 | -0.98207 | 0.155507 | -6.31528 | 2.7E-10 | 1.07E-07 |
| ALDH7A1 | 1109.392 | -0.98222 | 0.219492 | -4.47498 | 7.64E-06 | 0.000753 |
| XPA | 3772.446 | -0.9832 | 0.143954 | -6.83 | 8.49E-12 | 6.37E-09 |
| TRAPPC13 | 1074.835 | -0.98337 | 0.22941 | -4.28652 | 1.81E-05 | 0.001532 |
| CETN3 | 300.8882 | -0.98431 | 0.2987 | -3.29532 | 0.000983 | 0.04611 |
| ATG12 | 321.8563 | -0.9848 | 0.323265 | -3.04642 | 0.002316 | 0.093815 |
| COL4A3BP | 2148.772 | -0.98487 | 0.174199 | -5.65372 | 1.57E-08 | 3.79E-06 |
| TBCA | 1949.115 | -0.98516 | 0.129281 | -7.6203 | 2.53E-14 | 6.84E-11 |
| CDK7 | 954.6998 | -0.9854 | 0.197235 | -4.99609 | 5.85E-07 | 8.32E-05 |
| SMN1 | 484.8515 | -0.98668 | 0.257314 | -3.83454 | 0.000126 | 0.007689 |
| RASA1 | 2569.838 | -0.99166 | 0.157749 | -6.28632 | 3.25E-10 | 1.25E-07 |
| NNT | 831.0919 | -0.99215 | 0.277152 | -3.57982 | 0.000344 | 0.018285 |
| ISCA1 | 378.8438 | -0.99225 | 0.228829 | -4.3362 | 1.45E-05 | 0.001285 |
| TLE4 | 2053.262 | -0.99306 | 0.184894 | -5.37096 | 7.83E-08 | 1.48E-05 |
| SMC5 | 2298.131 | -0.99672 | 0.185991 | -5.35898 | 8.37E-08 | 1.53E-05 |
| ZNF462 | 3659.598 | -1.00208 | 0.203386 | -4.92699 | 8.35E-07 | 0.00011 |
| ARSK | 860.3221 | -1.00505 | 0.235041 | -4.27607 | 1.9E-05 | 0.001557 |
| RAD23B | 2861.157 | -1.00508 | 0.19344 | -5.19584 | 2.04E-07 | 3.18E-05 |
| FAM151B | 852.8956 | -1.00839 | 0.228549 | -4.41212 | 1.02E-05 | 0.00096 |
| KIAA1328 | 978.0536 | -1.0107 | 0.296842 | -3.40485 | 0.000662 | 0.032636 |
| GNE | 2906.738 | -1.01365 | 0.15659 | -6.47324 | 9.59E-11 | 4.63E-08 |
| WDR70 | 2089.651 | -1.01635 | 0.187668 | -5.41571 | 6.1E-08 | 1.18E-05 |
| RAD1 | 429.3684 | -1.01734 | 0.285174 | -3.56743 | 0.00036 | 0.019022 |
| MCC | 8662.181 | -1.01772 | 0.143249 | -7.10454 | 1.21E-12 | 1.48E-09 |
| SEMA6A | 1414.99 | -1.01811 | 0.31874 | -3.19418 | 0.001402 | 0.061903 |
| CLTA | 944.1712 | -1.01847 | 0.214183 | -4.75516 | 1.98E-06 | 0.000231 |
| DTWD2 | 1235.147 | -1.01881 | 0.23886 | -4.26532 | 2E-05 | 0.001624 |
| PTCD2 | 387.3273 | -1.02011 | 0.31959 | -3.19193 | 0.001413 | 0.062183 |
| LNPEP | 3259.078 | -1.02028 | 0.141313 | -7.21999 | 5.2E-13 | 7.24E-10 |
| GHR | 2007.978 | -1.0222 | 0.264497 | -3.86467 | 0.000111 | 0.006924 |
| NAA35 | 1596.897 | -1.02258 | 0.221967 | -4.60689 | 4.09E-06 | 0.000431 |
| DCP2 | 883.1796 | -1.02893 | 0.211469 | -4.86562 | 1.14E-06 | 0.000143 |
| FOCAD | 978.2924 | -1.02957 | 0.186789 | -5.51195 | 3.55E-08 | 7.29E-06 |
| C5orf34 | 817.6938 | -1.03091 | 0.276588 | -3.72725 | 0.000194 | 0.011266 |
| GPBP1 | 1164.349 | -1.03392 | 0.238857 | -4.32861 | 1.5E-05 | 0.001308 |
| MTAP | 311.8228 | -1.0347 | 0.245343 | -4.21735 | 2.47E-05 | 0.00193 |
| TXN | 9837.733 | -1.03682 | 0.281585 | -3.68208 | 0.000231 | 0.012913 |
| SPTLC1 | 1027.333 | -1.04189 | 0.17076 | -6.1015 | 1.05E-09 | 3.38E-07 |
| NDUFS4 | 1850.249 | -1.04312 | 0.180832 | -5.76846 | 8E-09 | 2.04E-06 |
| NFIB | 5875.974 | -1.04574 | 0.271149 | -3.85668 | 0.000115 | 0.007122 |
| IDUA | 1672.43 | -1.04674 | 0.261801 | -3.99822 | 6.38E-05 | 0.004421 |
| LMBRD2 | 1449.626 | -1.04834 | 0.153373 | -6.83521 | 8.19E-12 | 6.37E-09 |
| ANXA1 | 6063.822 | -1.05187 | 0.240641 | -4.37111 | 1.24E-05 | 0.001136 |
| SECISBP2 | 1465.895 | -1.0546 | 0.174616 | -6.03956 | 1.55E-09 | 4.74E-07 |
| SGTB | 774.454 | -1.05638 | 0.245524 | -4.30258 | 1.69E-05 | 0.001443 |
| POLR1E | 500.5549 | -1.05688 | 0.270912 | -3.90119 | 9.57E-05 | 0.006042 |
| RPS6 | 12018.69 | -1.05927 | 0.161216 | -6.57049 | 5.01E-11 | 2.69E-08 |
| PDZD2 | 4424.177 | -1.06019 | 0.210263 | -5.04221 | 4.6E-07 | 6.76E-05 |
| NDUFAF2 | 1074.647 | -1.06173 | 0.165334 | -6.42172 | 1.35E-10 | 6.07E-08 |
| RFX3 | 849.2942 | -1.06745 | 0.301356 | -3.54214 | 0.000397 | 0.020541 |
| DNAJC25 | 257.5531 | -1.06906 | 0.353593 | -3.02342 | 0.002499 | 0.097539 |
| KIAA2026 | 1575.552 | -1.07196 | 0.158957 | -6.74371 | 1.54E-11 | 9.93E-09 |
| TARS | 1035.756 | -1.07388 | 0.245851 | -4.36802 | 1.25E-05 | 0.001144 |
| DIMT1 | 303.186 | -1.0769 | 0.334801 | -3.21655 | 0.001297 | 0.05784 |
| MFSD7 | 2608.963 | -1.07853 | 0.236577 | -4.5589 | 5.14E-06 | 0.000522 |
| SMARCA2 | 4864.544 | -1.08212 | 0.155657 | -6.95196 | 3.6E-12 | 3.4E-09 |
| IKBKAP | 1536.876 | -1.08549 | 0.170803 | -6.35521 | 2.08E-10 | 8.52E-08 |
| RUSC2 | 637.7293 | -1.08877 | 0.31178 | -3.49213 | 0.000479 | 0.024334 |
| TOPORS | 438.8982 | -1.08952 | 0.308003 | -3.53737 | 0.000404 | 0.020757 |
| NLN | 1363.773 | -1.09218 | 0.174289 | -6.26646 | 3.69E-10 | 1.38E-07 |
| GNAQ | 3153.243 | -1.09389 | 0.174672 | -6.26254 | 3.79E-10 | 1.38E-07 |
| HAUS1 | 878.1838 | -1.09528 | 0.16512 | -6.63323 | 3.28E-11 | 1.93E-08 |
| BTF3 | 1950.21 | -1.09773 | 0.161015 | -6.81758 | 9.26E-12 | 6.58E-09 |
| CHSY3 | 2966.654 | -1.10027 | 0.288943 | -3.8079 | 0.00014 | 0.008451 |
| HMGCS1 | 755.9684 | -1.10112 | 0.362538 | -3.03725 | 0.002387 | 0.095339 |
| PIK3C3 | 2093.435 | -1.10351 | 0.158889 | -6.94514 | 3.78E-12 | 3.4E-09 |
| APBA1 | 832.7077 | -1.10786 | 0.240386 | -4.60869 | 4.05E-06 | 0.000431 |
| RFK | 128.6338 | -1.10924 | 0.339462 | -3.26765 | 0.001084 | 0.049995 |
| RPL37 | 21849.34 | -1.11137 | 0.225746 | -4.92312 | 8.52E-07 | 0.000111 |
| PALM2 | 489.9798 | -1.11538 | 0.272539 | -4.09255 | 4.27E-05 | 0.003115 |
| ATP5I | 1383.007 | -1.11708 | 0.234247 | -4.76883 | 1.85E-06 | 0.00022 |
| nAChRa7 | 494.6829 | -1.11871 | 0.331444 | -3.37527 | 0.000737 | 0.035576 |
| NSA2 | 2010.246 | -1.11948 | 0.191319 | -5.85134 | 4.88E-09 | 1.32E-06 |
| CDC42SE2 | 623.3512 | -1.12218 | 0.271889 | -4.12737 | 3.67E-05 | 0.002754 |
| CDC14B | 512.3391 | -1.12336 | 0.233054 | -4.82017 | 1.43E-06 | 0.000175 |
| PIGG | 1634.687 | -1.12654 | 0.207305 | -5.43421 | 5.5E-08 | 1.08E-05 |
| CMYA5 | 331.1701 | -1.13015 | 0.362044 | -3.12159 | 0.001799 | 0.076168 |
| FKTN | 683.0061 | -1.13137 | 0.231371 | -4.88986 | 1.01E-06 | 0.000128 |
| RCL1 | 441.7169 | -1.1331 | 0.248732 | -4.55549 | 5.23E-06 | 0.000526 |
| CNTLN | 2027.652 | -1.13415 | 0.20358 | -5.571 | 2.53E-08 | 5.35E-06 |
| TRIM23 | 825.9446 | -1.14204 | 0.198479 | -5.75397 | 8.72E-09 | 2.17E-06 |
| KIAA0020 | 951.5232 | -1.1433 | 0.222204 | -5.14526 | 2.67E-07 | 3.97E-05 |
| EPB41L4A | 2981.637 | -1.1468 | 0.375325 | -3.05548 | 0.002247 | 0.0917 |
| CBWD1 | 1187.074 | -1.1513 | 0.243422 | -4.72966 | 2.25E-06 | 0.000257 |
| APTX | 591.1478 | -1.15352 | 0.312999 | -3.68539 | 0.000228 | 0.012799 |
| DNAJA1 | 1307.951 | -1.15391 | 0.271573 | -4.249 | 2.15E-05 | 0.001726 |
| COX7C | 1551.841 | -1.15736 | 0.267137 | -4.33245 | 1.47E-05 | 0.001293 |
| TLE1 | 2923.503 | -1.1601 | 0.300976 | -3.85446 | 0.000116 | 0.007154 |
| **BCL11A** | **367.9346** | **-1.16084** | **0.360751** | **-3.21785** | **0.001292** | **0.057768** |
| ADAMTSL1 | 3628.515 | -1.16808 | 0.208231 | -5.60954 | 2.03E-08 | 4.64E-06 |
| **CEP250** | **327.7764** | **-1.17094** | **0.267091** | **-4.38406** | **1.16E-05** | **0.001085** |
| KIAA1328 | 264.4261 | -1.17346 | 0.337552 | -3.4764 | 0.000508 | 0.025615 |
| ERCC8 | 301.5397 | -1.17734 | 0.275278 | -4.27693 | 1.89E-05 | 0.001557 |
| CTIF | 1448.949 | -1.17886 | 0.301371 | -3.91165 | 9.17E-05 | 0.005897 |
| RPL17 | 10518.77 | -1.18195 | 0.198338 | -5.95926 | 2.53E-09 | 7.44E-07 |
| DEPDC1B | 427.6197 | -1.18415 | 0.362599 | -3.26572 | 0.001092 | 0.050166 |
| TPGS2 | 298.3752 | -1.1882 | 0.374169 | -3.17556 | 0.001495 | 0.065374 |
| FOCAD | 2475.702 | -1.18869 | 0.164733 | -7.21589 | 5.36E-13 | 7.24E-10 |
| HEXB | 2162.198 | -1.20459 | 0.199812 | -6.02858 | 1.65E-09 | 4.96E-07 |
| ERCC8 | 1090.214 | -1.2051 | 0.289439 | -4.16357 | 3.13E-05 | 0.002391 |
| HINT1 | 860.6018 | -1.20684 | 0.272246 | -4.4329 | 9.3E-06 | 0.000897 |
| PTGR1 | 1407.717 | -1.20923 | 0.356504 | -3.39191 | 0.000694 | 0.033842 |
| RNF38 | 3272.645 | -1.21075 | 0.25107 | -4.82237 | 1.42E-06 | 0.000174 |
| MOCS2 | 407.5173 | -1.21496 | 0.383043 | -3.17186 | 0.001515 | 0.065576 |
| NTRK2 | 5645.942 | -1.22141 | 0.387409 | -3.15277 | 0.001617 | 0.069135 |
| TTC37 | 1329.765 | -1.22186 | 0.117665 | -10.3843 | 2.92E-25 | 1.97E-21 |
| CEP250 | 622.4742 | -1.22747 | 0.300253 | -4.08814 | 4.35E-05 | 0.003158 |
| SEMA4D | 2258.962 | -1.22928 | 0.208774 | -5.88808 | 3.91E-09 | 1.1E-06 |
| INIP | 720.0572 | -1.23182 | 0.212243 | -5.80379 | 6.48E-09 | 1.68E-06 |
| PARP8 | 1078.837 | -1.23681 | 0.298548 | -4.14275 | 3.43E-05 | 0.00259 |
| OXCT1 | 1921.908 | -1.24143 | 0.204355 | -6.07488 | 1.24E-09 | 3.9E-07 |
| DAPK1 | 3221.179 | -1.2446 | 0.175504 | -7.09156 | 1.33E-12 | 1.49E-09 |
| C9orf85 | 1195.186 | -1.25302 | 0.224075 | -5.59199 | 2.24E-08 | 4.87E-06 |
| HIATL1 | 1013.095 | -1.25859 | 0.225133 | -5.59044 | 2.26E-08 | 4.87E-06 |
| FEM1C | 852.6827 | -1.26621 | 0.298622 | -4.24017 | 2.23E-05 | 0.001785 |
| ANKDD1B | 264.5658 | -1.27975 | 0.322051 | -3.97374 | 7.08E-05 | 0.004708 |
| RNF165 | 481.8556 | -1.29876 | 0.353406 | -3.67497 | 0.000238 | 0.013223 |
| SMC2 | 1068.814 | -1.30877 | 0.337665 | -3.87595 | 0.000106 | 0.006642 |
| MRPS36 | 404.8755 | -1.3166 | 0.276986 | -4.7533 | 2E-06 | 0.000231 |
| RNF20 | 1238.914 | -1.32173 | 0.298876 | -4.42235 | 9.76E-06 | 0.000929 |
| MRPL50 | 338.7392 | -1.35262 | 0.294993 | -4.58527 | 4.53E-06 | 0.000464 |
| ANKRD31 | 541.1271 | -1.36428 | 0.262099 | -5.2052 | 1.94E-07 | 3.08E-05 |
| UGCG | 1107.533 | -1.36802 | 0.276407 | -4.94927 | 7.45E-07 | 0.0001 |
| DEPDC1B | 840.5729 | -1.36885 | 0.420328 | -3.25663 | 0.001127 | 0.051543 |
| LHFPL2 | 460.9477 | -1.37919 | 0.296014 | -4.65921 | 3.17E-06 | 0.000349 |
| CENPK | 230.7583 | -1.38525 | 0.367613 | -3.76823 | 0.000164 | 0.00974 |
| FAM219A | 2126.557 | -1.38977 | 0.29629 | -4.69055 | 2.72E-06 | 0.000302 |
| SLC44A1 | 653.0707 | -1.39652 | 0.283426 | -4.92729 | 8.34E-07 | 0.00011 |
| SPTLC1 | 1140.313 | -1.4318 | 0.321006 | -4.46037 | 8.18E-06 | 0.000795 |
| ADAMTS19 | 749.9956 | -1.43836 | 0.431083 | -3.33662 | 0.000848 | 0.040336 |
| DCTN3 | 586.4481 | -1.49268 | 0.234482 | -6.36586 | 1.94E-10 | 8.41E-08 |
| LURAP1L | 1137.832 | -1.49607 | 0.364658 | -4.10266 | 4.08E-05 | 0.003027 |
| CAAP1 | 249.2649 | -1.5209 | 0.464767 | -3.2724 | 0.001066 | 0.049331 |
| POLR3C | 80.46687 | -1.52149 | 0.448719 | -3.39073 | 0.000697 | 0.033842 |
| PTCH1 | 2377.319 | -1.54715 | 0.363323 | -4.25832 | 2.06E-05 | 0.001666 |
| EMC4 | 137.589 | -1.58729 | 0.471543 | -3.36616 | 0.000762 | 0.036641 |
| PSD3 | 161.627 | -1.60911 | 0.519041 | -3.10015 | 0.001934 | 0.081648 |
| CA9 | 206.4407 | -1.61649 | 0.463875 | -3.48476 | 0.000493 | 0.02492 |
| RNF170 | 276.8493 | -1.62709 | 0.437464 | -3.71937 | 0.0002 | 0.011516 |
| **EFEMP1** | **951.4868** | **-1.6815** | **0.553833** | **-3.03611** | **0.002397** | **0.095339** |
| IL7R | 412.203 | -1.68465 | 0.386562 | -4.35804 | 1.31E-05 | 0.00119 |
| **ATP6V1C2** | **107.1363** | **-1.71934** | **0.562506** | **-3.05657** | **0.002239** | **0.091645** |
| C5orf51 | 217.0934 | -1.72662 | 0.426777 | -4.04572 | 5.22E-05 | 0.003708 |
| PSAT1 | 232.5785 | -1.834 | 0.539094 | -3.402 | 0.000669 | 0.032858 |
| **ACSF2** | **302.971** | **-1.85811** | **0.512172** | **-3.6279** | **0.000286** | **0.015545** |
| KLF9 | 131.8482 | -1.86754 | 0.575193 | -3.2468 | 0.001167 | 0.052788 |
| MOB3B | 693.9041 | -1.87505 | 0.383519 | -4.88907 | 1.01E-06 | 0.000128 |
| RGMB | 68.00297 | -1.91937 | 0.581379 | -3.30141 | 0.000962 | 0.045278 |
| MOB3B | 35.33287 | -2.01169 | 0.617819 | -3.25612 | 0.001129 | 0.051543 |
| KLF4 | 206.5577 | -2.09174 | 0.510124 | -4.10045 | 4.12E-05 | 0.003027 |
| **SELENBP1** | **197.9012** | **-2.18843** | **0.713876** | **-3.06555** | **0.002173** | **0.089205** |
| PSD3 | 578.2223 | -2.22216 | 0.445669 | -4.98612 | 6.16E-07 | 8.67E-05 |
| LOXHD1 | 188.8419 | -2.3035 | 0.646556 | -3.56273 | 0.000367 | 0.019291 |
| CCL28 | 55.13921 | -2.53497 | 0.732957 | -3.45856 | 0.000543 | 0.027271 |
| **EEF2** | **45.16995** | **-2.74374** | **0.908872** | **-3.01884** | **0.002537** | **0.097931** |
| **WIF1** | **159.1401** | **-2.83658** | **0.787788** | **-3.60069** | **0.000317** | **0.016945** |
| **PTPRZ1** | **4198.423** | **-3.13582** | **0.985121** | **-3.18319** | **0.001457** | **0.063884** |
| POU2F3 | 371.673 | -3.31783 | 0.849139 | -3.90729 | 9.33E-05 | 0.005919 |
| **WFDC2** | **82.01004** | **-3.3617** | **0.947334** | **-3.54859** | **0.000387** | **0.0202** |
| MLANA | 146.5917 | -3.61144 | 0.837242 | -4.31349 | 1.61E-05 | 0.001391 |
| **ANKRD66** | **29.46728** | **-4.27481** | **1.407414** | **-3.03735** | **0.002387** | **0.095339** |
| IFNW1 | 35.22225 | -4.60342 | 1.254843 | -3.66853 | 0.000244 | 0.01345 |
| **FGF16** | **17.11608** | **-5.05483** | **1.672312** | **-3.02266** | **0.002506** | **0.097539** |
| **RAB38** | **34.68411** | **-5.49654** | **1.608686** | **-3.41679** | **0.000634** | **0.031353** |
| C14orf180 | 15.67726 | -6.59154 | 2.026593 | -3.25252 | 0.001144 | 0.052024 |
| TYRP1 | 17.92289 | -7.07074 | 1.684238 | -4.19819 | 2.69E-05 | 0.002089 |
| TYR | 24.90332 | -7.32991 | 2.427038 | -3.0201 | 0.002527 | 0.097803 |

Table S5 – Enriched pathways on differentially expressed genes on males, ranked by a combination of FDR (cut off > 0.05) and fold enrichment.

| **Enrichment FDR** | **nGenes** | **Pathway Genes** | **Fold Enrichment** | **Pathway** | **Genes** |
| --- | --- | --- | --- | --- | --- |
| 0,0166 | 2 | 6 | 45,72 | GO:0000109 nucleotide-excision repair complex | ERCC8; RAD23B |
| 0,0242 | 3 | 30 | 13,72 | GO:1990391 DNA repair complex | XRCC4; ERCC8; RAD23B |
| 0,0242 | 3 | 31 | 13,27 | GO:0042470 melanosome | TYR; MLANA; TYRP1 |
| 0,0242 | 3 | 31 | 13,27 | GO:0048770 pigment granule | TYR; MLANA; TYRP1 |

Table S6 – Alternative exon usage identified between sexes and colour morphs

| ***sex*** | | | | | | | | | | |
| --- | --- | --- | --- | --- | --- | --- | --- | --- | --- | --- |
| *featureID* |  | *exonBaseMean* | *dispersion* | *stat* | *pvalue* | *padj* | *female* | *male* | *log2fold_male_female* | *gene_name* |
| E012 |  | 4,91 | 0,39 | 26,12 | 0,00 | 0,05 | 0,90 | 2,90 | 3,42 | SLC12A1 |
| ***colour*** | | | | | | | | | | |
| *featureID* |  | *exonBaseMean* | *dispersion* | *stat* | *pvalue* | *padj* | *brown* | *grey* | *log2fold_grey_brown* | *gene_name* |
| E007 |  | 19,76 | 0,06 | 24,80 | 0,00 | 0,09 | 5,27 | 3,70 | -1,12 | CD151 |
| E010 |  | 22,98 | 0,08 | 23,51 | 0,00 | 0,09 | 5,57 | 3,67 | -1,32 | DKC1 |
| E013 |  | 88,11 | 0,02 | 22,88 | 0,00 | 0,09 | 9,26 | 8,04 | -0,55 | EPS8L2 |

Table S7 – Number of SNPs retained after each filter step

| ***Filtering step*** | ***number SNPs*** |
| --- | --- |
| Call after GATK pipeline | 2506643 |
| Unique SNPs per loci | 1624691 |
| After custom filtration | 727994 |
| Only biallelic loci | 719512 |
| After HWE; MAF, and missing data | 65222 |

Table S8 – SNPs outliers (F_ST_) when factoring for sex. The * denotes significant results for prior odds = 100

| ***region*** | ***SNP*** | ***Gene*** | ***F_ST_*** | ***q_value*** | ***mapping*** |
| --- | --- | --- | --- | --- | --- |
|  |  |  |  |  |  |
| contig_323 | 4243292 | GHR | 0.21379 | 0.002* | Z |
| contig_835 | 5287480 | GSK3B | 0.19854 | 0.0299* | 5 |
| contig_1190 | 3508062 | COL4A3BP | 0.14777 | 0.032 | Z |
| contig_1205 | 1983000 | NCKAP1 | 0.14103 | 0.0356 | 7 |
| contig_1209 | 582327 | BMPR2 | 0.1653 | 0.0061 | 7 |
| contig_1209 | 622308 | BMPR2 | 0.14473 | 0.0267 | 7 |
| contig_1357 | 5179933 | PLEKHA5 | 0.17499 | 0.012 | 1 |
| contig_1563 | 136937 | GTF2H2 | 0.17681 | 0.012 | Z |
| contig_2710 | 9896507 | LEPR | 0.15141 | 0.0286 | 8 |
| contig_2710 | 9896529 | LEPR | 0.15155 | 0.0267 | 8 |
| contig_323 | 4243332 | GHR | 0.14233 | 0.0481 | Z |
| contig_331 | 16919082 | RAD23B | 0.12632 | 0.0423 | Z |
| contig_62 | 15622327 | GTSE1 | 0.15885 | 0.0213 | 1 |
| contig_835 | 5287480 | GSK3B | 0.19782 | 0.0016 | 5 |
| scaffold_47 | 9327707 | TTC14 | 0.1653 | 0.0304 | 9 |
| scaffold_487 | 41555103 | BBS9 | 0.16409 | 0.0174 | 2 |

Table S9 – SNPs outliers (F_ST_) when factoring for colour morph. The * denotes significant results for prior odds = 100

| ***region*** | ***SNP*** | ***Gene*** | ***F_ST_*** | ***q_value*** | ***mapping*** |
| --- | --- | --- | --- | --- | --- |
| contig_1357 | 2918047 | C2CD5 | 0.18648 | 0.001* | 1 |
| contig_1043 | 16630255 | FANCI | 0.15892 | 0.0236 | 10 |
| contig_331 | 6196881 | REEP5 | 0.12487 | 0.0306 | Z |
| contig_602 | 30809709 | BCKDHB | 0.15972 | 0.0058 | 3 |
| contig_63 | 6795672 | TDRD3 | 0.16603 | 0.0101 | 1 |
| scaffold_47 | 8918045 | MFN1 | 0.14 | 0.0464 | 9 |
| scaffold_487 | 9288408 | PDSS1 | 0.18163 | 0.0079 | 2 |
| scaffold_487 | 28752261 | JAZF1 | 0.15687 | 0.0161 | 2 |

| **Enrichment FDR** | **nGenes** | **Pathway Genes** | **Fold Enrichment** | **Pathway** | **Genes** |
| --- | --- | --- | --- | --- | --- |
| 0,0266 | 2 | 40 | 176,313 | GO:0032587 ruffle membrane | C2CD5, §EPS8L2 |
| 0,0462 | 1 | 3 | 1175,417 | GO:0035651 AP-3 adaptor complex binding | RAB38 |
| 0,04621 | 1 | 5 | 705,250 | GO:0090383 phagosome acidification | RAB38 |
| 0,04621 | 1 | 5 | 705,250 | GO:1903232 melanosome assembly | RAB38 |
| 0,04621 | 1 | 5 | 705,250 | GO:0035650 AP-1 adaptor complex binding | RAB38 |
| 0,04621 | 1 | 7 | 503,750 | ENSGALP00000029039 interacting protein | C2CD5 |
| 0,04621 | 1 | 7 | 503,750 | GO:0031340 positive reg. of vesicle fusion | C2CD5 |
| 0,04621 | 1 | 7 | 503,750 | GO:1900029 positive reg. of ruffle assembly | EPS8L2 |
| 0,04621 | 1 | 9 | 391,806 | GO:0035646 endosome to melanosome transport | RAB38 |
| 0,04621 | 1 | 9 | 391,806 | GO:0043476 pigment accumulation | RAB38 |
| 0,04621 | 1 | 9 | 391,806 | GO:0043482 cellular pigment accumulation | RAB38 |
| 0,04621 | 1 | 9 | 391,806 | GO:0043485 endosome to pigment granule transport | RAB38 |
| 0,04621 | 1 | 9 | 391,806 | GO:0048757 pigment granule maturation | RAB38 |
| 0,04621 | 1 | 9 | 391,806 | GO:0033162 melanosome membrane | RAB38 |
| 0,04621 | 1 | 9 | 391,806 | GO:0045009 chitosome | RAB38 |
| 0,04621 | 1 | 9 | 391,806 | GO:0090741 pigment granule membrane | RAB38 |
| 0,04621 | 1 | 10 | 352,625 | GO:0032426 stereocilium tip | EPS8L2 |
| 0,04621 | 1 | 11 | 320,568 | ENSGALP00000014320 interacting protein | FAM135A^+^ |
| 0,04621 | 1 | 12 | 293,854 | ENSGALP00000041058 interacting protein | FAM135A^+^ |
| 0,04621 | 1 | 13 | 271,250 | GO:0032438 melanosome organization | RAB38 |

Table S10 – Enriched pathways of all genes found to co-vary with colour morph – from this work and from Baltazar-Soares et al 2024 (^+^). Ranked by a combination of FDR (cut off > 0.05) and fold enrichment

**Supplemental Figures captions**

**Fig. S1 – Aviary weight measurements.** Weight has been measured in two time points, 15 days post hatching and 90 days post hatching. Here we present pairwise comparisons between sexes. Note that only significant interactions (t-test, p <0.05) are depicted in the picture. * p = 0.05, *** p < 0.01.

**Fig. S2 – MA plot for differentially expressed genes between sexes.** Distribution of log-fold changes across expressed genes. In grey, expressed genes that do not significantly differed in expression levels. In blue, differentially significantl expressed genes (positive y-axis for females and negative y-axis for males).

**Fig. S3 – DEGs among different groups:** Heatmap showing 352 differentially expressed genes between the sexes. Samples are organized through a hierarchical clustering approach and cluster well into females and males for the expression of the sex DEGs. Grey and brown colours on top of the heatmap indicate the morph type and purple colours on the left side of the plot distinguish the two gene groups clustered by similarity in expression. Blue colours group the genes by location into Z chromosome located, not Z chromosome located and not mapped to chicken genome. Z scores for expression differentiation are coloured along a blue – red colour gradient.

**Fig. S4 – Differential exon usage:** Read counts per exon are plotted to visualize differential exon usage in one gene linked with sex and three genes linked with morph. For each gene, we plotted expression (fitted count estimated from the glm regression), exon usage (fitted count estimates standardized for gene expression average to visualize the exon usage effect only) and the gene (exons in blocks, introns as lines) along the genomic region of the respective gene. Differentially used exons are indicated in purple and colours indicate the morph and sex types.

**Fig S5 – Network of GOterms identified among differentially expressed genes in males**
